# Evolving understanding of rumen methanogen ecophysiology

**DOI:** 10.3389/fmicb.2023.1296008

**Published:** 2023-11-06

**Authors:** Bela Haifa Khairunisa, Christian Heryakusuma, Kelechi Ike, Biswarup Mukhopadhyay, Dwi Susanti

**Affiliations:** ^1^Microbial Discovery Research, BiomEdit, Greenfield, IN, United States; ^2^Genetics, Bioinformatics, and Computational Biology, Virginia Tech, Blacksburg, VA, United States; ^3^Department of Biochemistry, Virginia Tech, Blacksburg, VA, United States; ^4^Department of Biology, North Carolina Agricultural and Technical State University, Greensboro, NC, United States; ^5^Virginia Tech Carilion School of Medicine, Virginia Tech, Blacksburg, VA, United States

**Keywords:** rumen, methanogen, methane, greenhouse gas, archaea, ruminants, microbiome

## Abstract

Production of methane by methanogenic archaea, or methanogens, in the rumen of ruminants is a thermodynamic necessity for microbial conversion of feed to volatile fatty acids, which are essential nutrients for the animals. On the other hand, methane is a greenhouse gas and its production causes energy loss for the animal. Accordingly, there are ongoing efforts toward developing effective strategies for mitigating methane emissions from ruminant livestock that require a detailed understanding of the diversity and ecophysiology of rumen methanogens. Rumen methanogens evolved from free-living autotrophic ancestors through genome streamlining involving gene loss and acquisition. The process yielded an oligotrophic lifestyle, and metabolically efficient and ecologically adapted descendants. This specialization poses serious challenges to the efforts of obtaining axenic cultures of rumen methanogens, and consequently, the information on their physiological properties remains in most part inferred from those of their non-rumen representatives. This review presents the current knowledge of rumen methanogens and their metabolic contributions to enteric methane production. It also identifies the respective critical gaps that need to be filled for aiding the efforts to mitigate methane emission from livestock operations and at the same time increasing the productivity in this critical agriculture sector.

## 1. Introduction

Livestock production in the US emitted close to 200 million metric tons of CO_2_-equivalent (MMT CO_2_–e) of methane, mainly originating from enteric fermentation in beef and dairy cattle representing 72 and 25% of emissions from livestock, respectively (EPA, 2022). The corresponding value at the global scale is approximately 2,500 MMT CO_2_-e (EPA, 2023a), and it is estimated to rise substantially due to an increase in demand for milk and meat to feed the 9.8 billion global population by 2050 (FAO, 2018; Henchion et al., 2021).

Methane is 28 times more potent greenhouse gas (GHG) with a much shorter shelf-life than CO_2_ (EPA, 2023b). In the rumen, it is produced as a by-product of microbial fermentation, and methanogenic archaea or methanogens are the only microorganisms that are known to produce methane anaerobically (Smith and Hungate, 1958; Ramanathan et al., 1985; Wolfe, 1992). In addition to contributing to global warming, methane emission from the rumen causes a loss of 2–12% of the energy provided by the feed (Johnson and Johnson, 1995; Janssen, 2010). Hence, a reduction of methane emission from cattle would have a greater near-term contribution to the effort toward mitigating global climate change and improving animal productivity (Janssen, 2010; Beauchemin et al., 2020).

For the above-mentioned importance, the metabolism of rumen microbes including methanogens has been investigated for almost eight decades (Barker, 1936; Elsden, 1945; Hungate, 1950; Beijer, 1952; Hungate, 1966; Henderson et al., 2015; Seshadri et al., 2018). These studies yielded a plethora of basic and applied science information about rumen methanogens including their role in facilitating microbial fermentation in the rumen (Hungate, 1966; Beauchemin et al., 2020). These details have been leveraged for developing tools for mitigating methane emission in the livestock industry and some of these can provide an average of 30% reduction in methane production with acceptable safety in both beef and dairy cattle (Yu et al., 2021). However, the outcome varies greatly (Patra et al., 2017; Arndt et al., 2022). What causes such variabilities? Which methanogens escape such intervention and how could one target them effectively? What factors drive the composition of a rumen methanogen community over another, spatially and temporally? Answering these questions requires a deeper understanding of the metabolic diversity and *in situ* physiology of rumen methanogens, which sorely remains incomplete even after close to eight decades of interrogation. It is because the current knowledge base for this field has mostly been built on studies with pure culture isolates from the rumen, which are a few, and inferences from the properties of non-rumen methanogen isolates (Jeyanathan, 2010; Seshadri et al., 2018). The technical hurdles of working with strict anaerobes and the absence of clues to specific auxotrophies have limited the isolation efforts, which could have allowed useful *in vitro* studies.

The culture-independent approaches leveraging high throughput omics are beginning to fill the above-mentioned gap in terms of phylogenetic diversity and metabolic potentials. The discovery of species from the *Methanomassiliicoccales* order that provide an additional route for removing the hydrogen-based thermodynamic block on ruminal fermentation (Borrel et al., 2013) and key genomic features that allow rumen methanogens to associate with other organisms (Leahy et al., 2010; Ng et al., 2016) and battle the toxicity of plant product (i.e., tannin) are examples of such advances (Kelly et al., 2016c; Loh et al., 2020). However, the absence of information on the metabolic and physiological properties of individual rumen methanogens that are generally obtained from studies on pure culture isolates or even low complexity enrichments has prevented making a clear sense of physiological data originating from *in vivo* or whole animal-based measurements.

**Figure 1:**
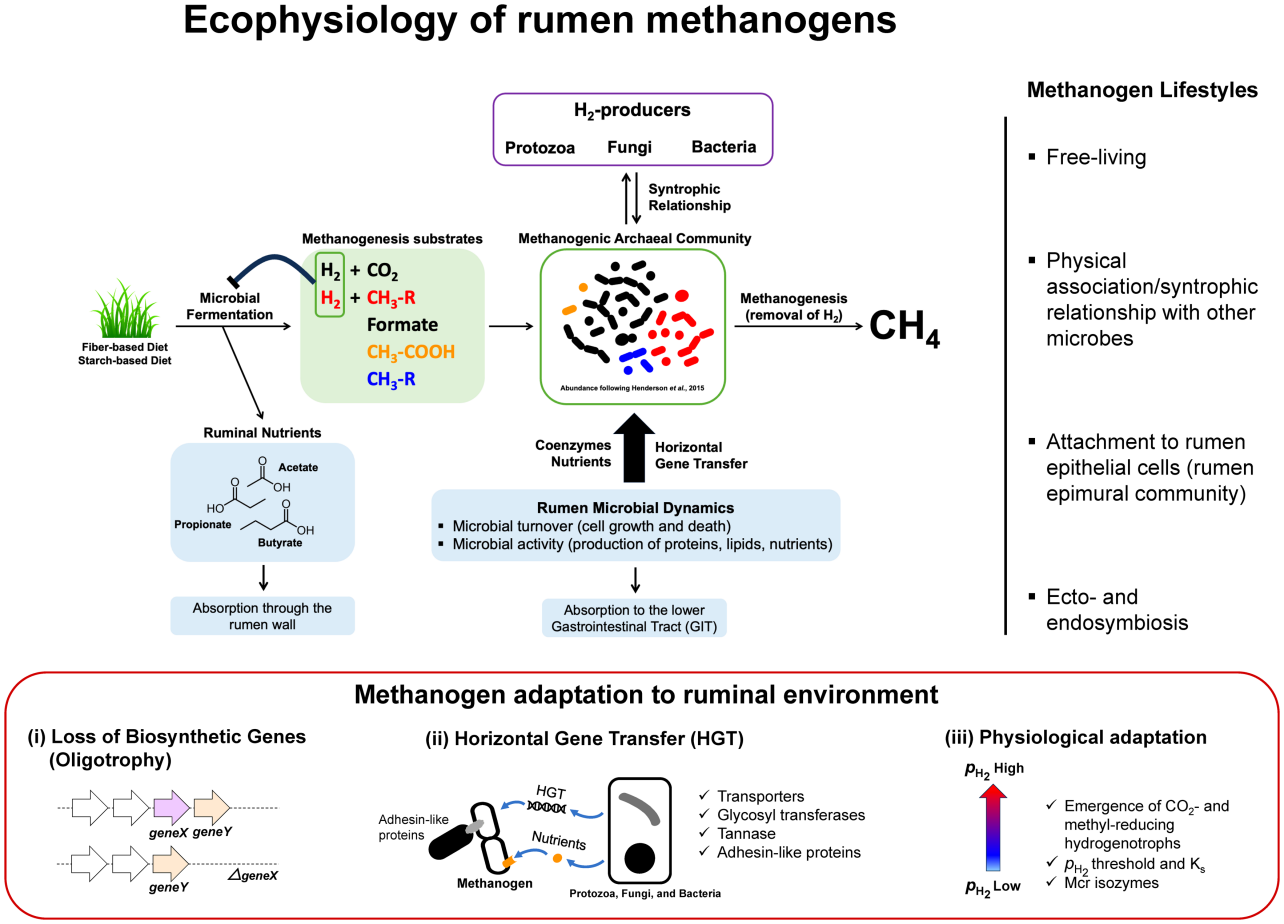
Ecophysiology and metabolic adaptation of rumen methanogens. A schematic diagram illustrating functional roles of methanogens that facilitate the continuation of rumen microbial fermentation by removal of H_2_ from microbial fermentation to generate methane. In the process, methanogens interact with different functional guilds via syntrophic associations and cross-feedings. Uptakes of nutrients and genetic materials via horizontal gene transfer (HGT) are shaping rumen methanogen metabolism, physiology, and lifestyle resulting in better adaptations and competitiveness in the rumen environment. Interactions between methanogens and other rumen microbiota are diverse and complex where methanogens are found as free-living, in a physical association or syntrophic relationship with other microbes, attach to the rumen epithelial cells as part of rumen epimural community, or ecto−/endosymbiosis with protozoa (right panel). Metabolic adaptation of methanogens in rumen environment (lower panel) results in loss of biosynthetic genes generating oligotrophy, acquisition of new functions through HGT, and physiological adaptation to methanogenic substrate fluctuations in the rumen (i.e., high and low $p_{H_{2}}$ conditions following feeding) that have significant impacts on the emergence of CO_2_- and methyl-reducing hydrogenotrophs (i.e., K_s_ and the deployment of different Mcr isozymes).

This review presents a summary and analysis of the past and evolving knowledge of rumen methanogens (Figure 1) including the ongoing and upcoming research that would fill the above-mentioned gaps and help the efforts to mitigate enteric methane emissions while bringing sustainability to the livestock industry.

## 2. Methanogenic archaea, a thermodynamic facilitator in rumen fermentation

**Figure 2:**
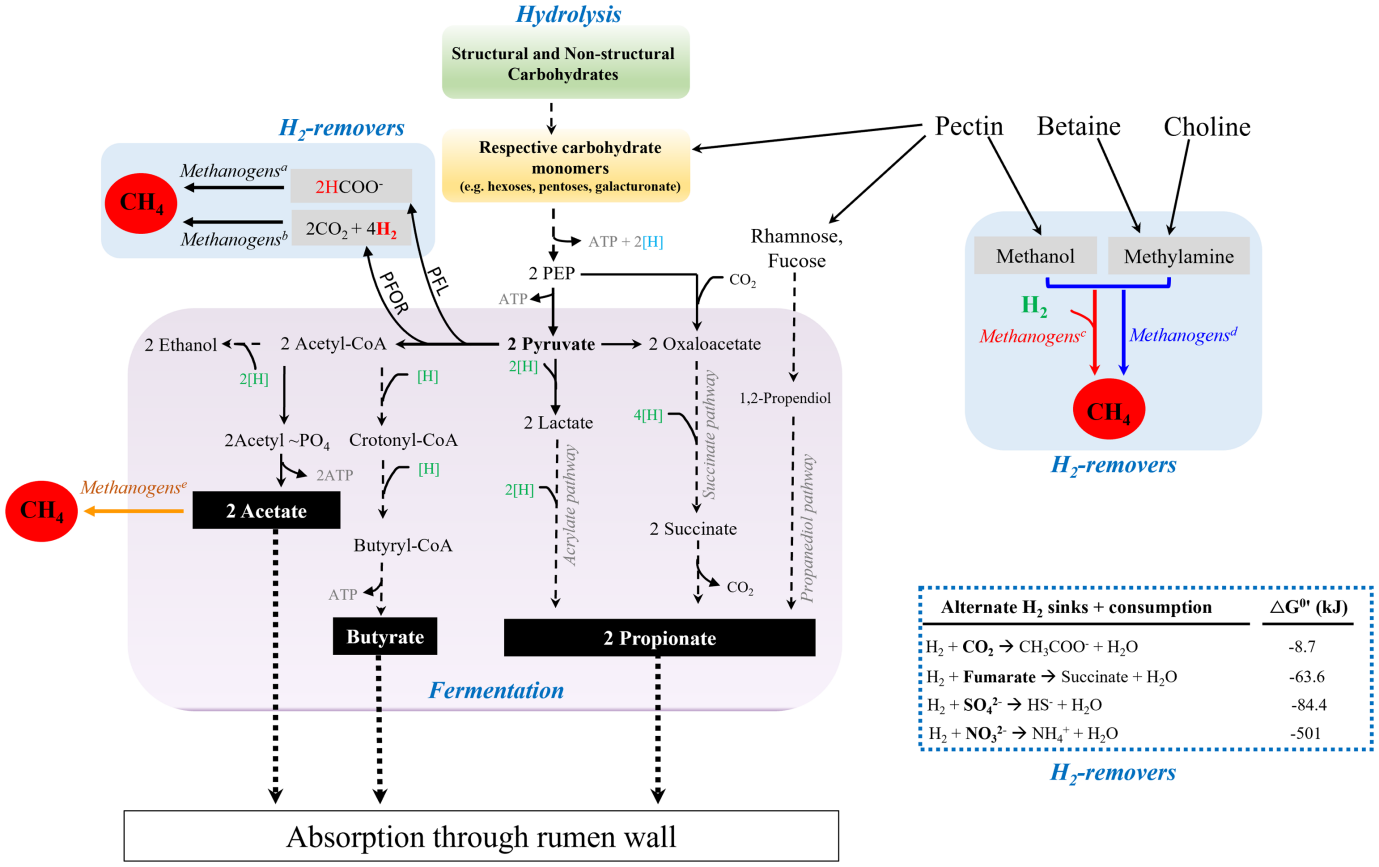
Carbohydrate degradation in the reticulorumen (rumen) of ruminants. Microbial degradation of structural carbohydrates and fermentation of resulting sugar monomers generate fatty acids, acetate, propionate, and butyrate. H_2_ is generated from the electron confurcation of NADH and Fdx_red_ during decarboxylation of pyruvate to generate acetyl-CoA. Generation of acetyl CoA from pyruvate can be performed by the actions of Pyruvate:Ferredoxin OxidoReductase (PFOR) or Pyruvate Formate Lyase (PFL). The H_2_ level is kept low by formate-dependent methanogenesis, CO_2_- and methyl-reducing hydrogenotrophs (superscripts a, b, and c, respectively), thus relieving the thermodynamic block on reoxidation of NADH and fermentation. Methyl-dismutating and acetoclastic methanogenesis are not commonly found in the rumen (superscripts d and e, respectively). When available, sulfate, nitrate, and fumarate can be used as alternate hydrogen sinks, blue dashed-lined box. Blue and green [H], production and consumption of reducing equivalent or (NAD(P)H), respectively; black dashed-lines, multi-step pathway; black solid lines, process in the rumen; black dotted-lines, absorption of volatile fatty acids by rumen wall.

Ruminants gain 70% of their energy from microbial activities that degrade feed materials in the first two compartments of the digestive tract, the rumen and the reticulum, which collectively called reticulorumen and hereafter is referred to as rumen (Flint and Bayer, 2008; Yeoman and White, 2014). The rumen microbial community is composed of bacteria, protozoa, archaea, and fungi in the order of the most abundant to the least (Hungate, 1966; Henderson et al., 2015; Seshadri et al., 2018); highly abundant and diverse virus populations are also important components in the rumen even though it has not been studied significantly (Gilbert et al., 2020). These individual members of rumen microbial community have been co-evolving with the ruminants for about 50 million years (Webb and Taylor, 1980; Hackmann and Spain, 2010; Jiang et al., 2014), making them resilient to environmental perturbation through their overlapping metabolic functionality (Weimer, 2015). Their concerted actions convert fermentable carbohydrates and amino acids anaerobically via fermentation into volatile fatty acids that provide energy to the animals and surplus reducing equivalents in the forms of hydrogen and formate, with most products coming from carbohydrates. If unutilized, excess H_2_ blocks the progress of fermentation thermodynamically, and in the rumen and many other anaerobic biodegradation systems, this block is removed by methanogens that utilize the excess H_2_ and generate methane (Figures 1, 2; Zinder, 1993).

Working synergistically, a group of bacteria, fungi, and protozoa hydrolyze cellulose and hemicellulose fibers into respective sugar monomers, and ferment these products into primarily three major volatile fatty acids, namely acetate, propionate, and butyrate that are absorbed by rumen epithelial walls (Hungate, 1966; Czerkawski and Breckenridge, 1973; Prins and van der Meer, 1976; Wolin, 1979; Williams and Coleman, 1997; Ragsdale, 2003; Sawers and Clark, 2004; Reichardt et al., 2014; Henderson et al., 2015; Hackmann et al., 2017; Gruninger et al., 2019; Ungerfeld, 2020; Williams et al., 2020; Pereira et al., 2022). In addition, lactate, ethanol, and succinate are produced as reduced intermediates (Gottschalk, 1986; Hackmann et al., 2017), where lactate and succinate are further converted to propionate (Gottschalk, 1986; Weimer, 1998; Reichardt et al., 2014; Hackmann et al., 2017; Moraïs and Mizrahi, 2019; Ungerfeld, 2020). Figure 2 summarizes this overall process. Acetate, propionate, and butyrate account for 40–75%, 15–40%, and 10–20% of the total rumen VFAs, respectively (Wolin, 1960; Bergman, 1990; DeFrain et al., 2004). Propionate serves as a major precursor for the biosynthesis of glucose through gluconeogenesis in the liver, which in turn is used as an energy source for the animal (Young, 1977). Acetate and butyrate can be used as precursors in lipid biogenesis by the host (Black et al., 1961; Hanson and Ballard, 1967; Moran, 2005).

Production of acetate and butyrate from glucose is associated with more negative ΔG^o^’ values than is propionate production (van Lingen et al., 2016). In addition, the production of propionate is associated with a net consumption of two moles of H_2_ per mole of glucose utilized, whereas that of acetate and butyrate lead to net productions of four and two moles of H_2_, respectively (van Lingen et al., 2016; Leahy et al., 2022). Accordingly, despite a higher thermodynamic feasibility of acetate and butyrate production from glucose under standard conditions, the generation of these VFAs is less favored as it leads to H_2_ accumulation and consequent thermodynamic inhibition of microbial fermentation.

The above-mentioned fermentation process generates pyruvate, ATP, and NADH (Figure 2). To allow unimpeded continuation of the fermentation process, NAD^+^ must be regenerated (Baldwin and Allison, 1983; Stams and Plugge, 2009). Depending on the prevailing cellular redox status (i.e., NAD^+^/NADH ratio) of the cells, it can be done through the production of reduced fermentation products such as ethanol, lactate, and propionate, and/or hydrogen generation via NADH:Ferredoxin oxidoreductase coupled with a hydrogenase or via electron confurcation reaction involving NADH and reduced ferredoxin (Fdx_red_) (Schut and Adams, 2009; Stams and Plugge, 2009). Processing of pyruvate via Pyruvate Formate Lyase (PFL) provides acetyl-CoA and formate, and the latter can be excreted or oxidized to H_2_ by formate hydrogen lyase (Baldwin and Allison, 1983, Stams and Plugge, 2009). As H_2_ accumulates, elevating its partial pressure or $p_{H_{2}}$ , it blocks NADH oxidation thermodynamically (Baldwin and Allison, 1983; Gottschalk, 1986; Stams and Plugge, 2009); thus, H_2_ is a central regulator and called the ‘currency’ of rumen fermentation (Czerkawski, 1986). Hydrogenotrophic methanogens remove this block on fermentation by consuming H_2_ via CO_2_ and methyl group reduction to methane (4H_2_ + CO_2_ → CH_4_ + 2H_2_O; H_2_ + CH_3_-X → CH_4_ + HX) and allowing NAD^+^ regeneration (Baldwin and Allison, 1983; Stams and Plugge, 2009). Excretion of formate, as mentioned above, lowers pH and its sequential oxidation to H_2_ imposes a thermodynamic block and methanogens alleviate these problems via formate methanogenesis (4HCOO^−^ + 4H^+^ → CH_4_ + 3CO_2_ + 2H_2_O) (Figure 2).

The process of electron transfer from a hydrogen producer to a methanogen via hydrogen as a vehicle was the first recognized case of interspecies electron transfer (IET) (Bryant et al., 1967). Direct IET (DIET) occurring via conducting pili or nanowires, or IET employing extracellular cytochromes that occur in other ecological systems (Lovley and Holmes, 2022) remains to be investigated for rumen microbiome (Kelly et al., 2022). With fiber digestion by protozoa, a unique reductant transfer process is seen. Here, protozoa release excess reductant as H_2_ through hydrogenosome, a mitochondria-type organelle representing an ancient bacterial endosymbiont (Lewis et al., 2020), which is captured directly by methanogens living syntrophically as protozoal endo- and ecto-symbiont (Vogels et al., 1980; Stumm and Zwart, 1986; Belanche et al., 2014). These symbiotic methanogens representing 10–20% of rumen methanogens contribute to 15–35% of ruminal methane production (Hegarty, 1999; Morgavi et al., 2008, 2012). This association is non-specific in terms of a methanogen’s selectivity for protozoa type (Henderson et al., 2015).

Figure 2 shows alternate routes for hydrogen removal in the rumen with the respective thermodynamic potentials. Except for acetogenesis (4H_2_ + 2CO_2_ → CH_3_COO^−^ + 2H_2_O + H^+^), which utilizes readily available CO_2_, these alternate avenues are used only if the respective electron acceptors are available in the rumen. For example, the sulfate reduction pathway occurs only when the sulfate concentration in the rumen is sufficient (Huisingh et al., 1974).

## 3. Expanding concepts of rumen methanogens’ diversity, physiology, and metabolism

Methanogens account for less than 3.3% of the total rRNA gene sequences in bovine rumen (Patra et al., 2017) and the dominant rumen methanogens are rather conserved across geographical regions (Henderson et al., 2015). Despite this relatively low abundance, methanogens have a major impact on microbial metabolism in this ecosystem for the reasons mentioned above. In this section, the diversity and methanogenesis or energy conservation processes of rumen methanogens are summarized and discussed.

### 3.1. Diversity

According to the taxonomic classification of the Genome Taxonomy Database (GTDB; Parks et al., 2022), the methanogen phyla represented in the rumen microbiome are Halobacteriota (H), Methanobacteriota (M), and Thermoplasmatota (T) (Janssen and Kirs, 2008; Henderson et al., 2015; Parks et al., 2022). These methanogens belong to four orders (phyla): *Methanobacteriales* (M), *Methanomicrobiales* (H), *Methanosarcinales* (H), *Methanomassiliicoccales* (T). The reports of *Methanococcales* (M) especially from *Methanocaldococcaceae* family and *Methanopyrales* (M) phyla, representing hyperthermophiles, in rumen samples (Janssen and Kirs, 2008; Henderson et al., 2015; Tan et al., 2021) are likely artifactual, and *Methanocellales* (H) have never been found in rumen. The identification of *Methanomassiliicoccales* in the rumen as major utilizers of hydrogen via a non-CO_2_ reduction route reshaped the concept of hydrogenotrophy (Borrel et al., 2013, 2014; Li et al., 2016; Kelly et al., 2016a,b; Garcia et al., 2022).

The rumen methanogen community is dominated by members of *Methanobacteriales*, especially from *Methanobrevibacter* and *Methanosphaera* genera, and those of *Methanomassiliicoccales*, with small contributions from *Methanomicrobium* and *Methanosarcina* genera (Henderson et al., 2015). *Methanobrevibacter gottschalkii*, *Methanobrevibacter ruminantium*, *Methanosphaera* sp., and two *Methanomassiliicoccaceae* (formerly grouped as the rumen cluster C or RCC) comprise close to 90% of the total rumen methanogen rRNA gene sequences with *Methanobrevibacter* covering 74% of the total sequences and the rest 16% belonging to *Methanosphaera* sp. and *Methanomassiliicoccaceae* (Janssen and Kirs, 2008; Henderson et al., 2015). These abundance values, however, are dynamic and vary across hosts and diets, even though the core methanogen players are rather conserved (Henderson et al., 2015). Table 1 describes all known pure culture isolates of rumen methanogens and their key cellular characteristics. Some of these features are discussed below; energy metabolism is covered in Section 3.2.

**Table 1:** Growth and nutritional requirements of select rumen-associated methanogens.

Methanogen species (culture depository)	Strain designation, isolated from	Substrate(s) for CH_4_ production [growth factor(s)]	Cell wall types	Optimum pH, T (°C)	Doubling time (h), medium, substrate	Lifestyle (endosymbiont, ectosymbiont, syntrophs)	GenBank ID, Gold project ID, Gold analysis project ID, Hungate1000 collection number	References
*Methanobrevibacter* spp. [SGMT and RO Clade (King et al., 2011)]								
*Methanobrevibacter smithii* (DSM 861, ATCC 35061)	PS, anaerobic sewage digester	H_2_ + CO_2_, formate [stimulatory – acetate]	PM	6.9–7.4, 39	6.7, BRN, H_2_/CO_2_ (80:20) 12.7, BRN, formate (290 mM)	[potentially] ectosymbiont through the production of adhesin-like protein	GCA_000016525, Gp0000134, Ga0029348, NA	Balch et al. (1979), Rea et al. (2007), Samuel et al. (2007), and Ng et al. (2016)
*Methanobrevibacter smithii* (DSM 2374)	F1, human feces	H_2_ + CO_2_, formate [required – trypticase, yeast extract]	PM	ND	ND	NA	GCA_000151225, Gp0003674, Ga0029349, NA	Miller et al. (1982)
*Methanobrevibacter smithii* (DSM 2375)	ALI, human large intestine	NR [NR]	NR	ND	ND	NA	GCA_000151245, Gp0003638, Ga0029350, NA	Miller and Wolin (1981) and Miller et al. (1982)
*Methanobrevibacter gottschalkii* (DSM 11977)	HO, horse feces	H_2_ + CO_2_ [required – acetate, yeast extract, trypticase peptone]	PM	7.0, 37	ND	[potentially] ectosymbiont through the production of adhesin-like protein	GCA_003814835, Gp0290545, Ga0244664, NonHun83	Miller and Lin (2002) and Ng et al. (2016)
*Methanobrevibacter gottschalkii* (DSM 11978)	PG, pig feces	H_2_ + CO_2_ [NR]	PM	NR	ND	NA	GCA_900109595, Gp0127403, Ga0104357, HUN396	Lin and Miller (1998)
*Methanobrevibacter millerae* (DSM 16643)	ZA-10, bovine rumen	H_2_ + CO_2_, formate [required – acetate, yeast extract, trypticase peptone] [stimulatory – valerate, isovalerate, 2-methylbutyrate, isobutyrate]	PM	7.0–8.0, 39	5.4, BRN, H_2_/CO_2_ (80:20) 14.6, BRN, formate (150 mM)	[potentially] ectosymbiont through the production of adhesin-like protein	GCA_900103415, Gp0087971, Ga0007632, HUN273	Rea et al. (2007) and Ng et al. (2016)
*Methanobrevibacter millerae* (NA)	SM9, sheep rumen	H_2_ + CO_2_, formate [required – acetate, yeast extract, trypticase peptone]	PM	ND	ND	[potentially] ectosymbiont through the production of adhesin-like protein	GCA_001477655, Gp0007703, Ga0104112, NonHun84	Kelly et al. (2016c) and Ng et al. (2016)
*Methanobrevibacter thaurei* (DSM 11995)	CW, cattle feces	H_2_ + CO_2_ [required – acetate, yeast extract, trypticase peptone]	PM	7.0, 37	ND	[potentially] ectosymbiont through the production of adhesin-like protein	GCA_003111625, Gp0113775, Ga0074444, NonHun89	Miller and Lin (2002) and Ng et al. (2016)
*Methanobrevibacter ruminantium* (DSM 1093, ATCC 35063)	M1, cattle rumen	H_2_ + CO_2_, formate [required – acetate, 2-methylbutyrate, amino acids (most stimulating T, H, M), coenzyme M]	PM	6.3–6.8, 39	16.8, BRN, H_2_/CO_2_ (80:20)29.4, BRN, formate (150 mM)	Ectosymbiont of protozoa of genera *Epidinium* and *Endodinium*, and with H_2_-producing bacteria *Butyrivibrio proclasticus* through the production of an adhesin-like protein	GCA_000024185, Gp0002311, Ga0029347, NonHun86	Bryant et al. (1971), Balch and Wolfe (1976), Balch et al. (1979), Rea et al. (2007), Leahy et al. (2010), and Ng et al. (2016)
*Methanobrevibacter olleyae* (DSM 16632)	KM1H5-1P, sheep rumen	H_2_ + CO_2_, formate [required – acetate]	PM	7.5, 39	14.5, BRN, H_2_/CO_2_ (80:20)15.3, BRN, formate (220 mM)	[potentially] ectosymbiont through the production of adhesin-like protein	GCA_900114585, Gp0087972, Ga0007633, HUN274	Rea et al. (2007) and Ng et al. (2016)
*Methanobrevibacter boviskoreani* (DSM 25824)	JH1, cattle rumen	H_2_ + CO_2_, formate [required – yeast extract, coenzyme M, and fatty acids (valerate, isovalerate, 2-methylbutyrate, isobutyrate)]	PM	6.5–7.0, 37–40	ND	NA	GCA_000320505, Gp0035818, Ga0021326, NonHun82	Lee et al. (2013)
*Methanobrevibacter woesei* (DSM 11979)	GS, goose feces	H_2_ + CO_2_, formate [required – acetate, yeast extract, trypticase peptone]	PM	7.0, 37	ND	NA	GCA_003111605, Gp0113776, Ga0074445, NonHun90	Miller and Lin (2002)
*Methanobrevibacter wolinii* (DSM 11976)	SH, sheep feces	H_2_ + CO_2_ [required – acetate, yeast extract, trypticase peptone, coenzyme M, and fatty acids (valerate, isovalerate, 2-methylbutyrate, isobutyrate)]	PM	7.0, 37	ND	NA	GCA_000621965, Gp0047017, Ga0005592, HUN166	Miller and Lin (2002)
*Methanosphaera* spp.								
*Methanosphaera stadtmanae* (DSM 3091, ATCC 43021)	MCB-3, human feces	H_2_ + methanol [required – acetate, amino acids (L, I), thiamin] [stimulatory – biotin]	PM	6.5–6.9, 36–40	ND	[potentially] ectosymbiont through the production of adhesin-like protein	GCA_000012545, Gp0000406, Ga0029374, NA	Miller and Wolin (1985) and Ng et al. (2016)
*Methanosphaera stadtmanae* (NA)	ISO3-F5, sheep rumen	H_2_ + methanol [required – acetate, yeast extract] [stimulatory – fatty acids (valerate, isovalerate, 2-methylbutyrate, butyrate, isobutyrate, propionate)]	PM	6.7–6.8, 39	ND	NA	NA	Jeyanathan (2010)
*Methanosphaera stadtmanae* (NA)	BMS, bovine rumen	H_2_ + methanol [required –yeast extract, casein hydrolysate, rumen fluid]	PM	6.7, 37	ND	NA	GCA_003268005, Gp0119560, Ga0105677, NA	Hoedt et al. (2018)
*Methanomicrobium* spp.								
*Methanomicrobium mobile* (DSM 1539, ATCC 35094)	BP, cattle rumen	H_2_ + CO_2_, formate [required – yeast extract or vitamin-free casamino acid, acetate, isovalerate, 2-methylbutyrate, isobutyrate, indole, pyridoxine, thiamine, biotin, cobalamin, PABA, boiled cell extract of *Methanothermobacter thermautotrophicus*, coenzyme B]	RS-layer	6.1–6.9, 40	ND	Found in association with protozoa of genera *Entodinium*, *Metadinium*, and *Ophryoscolex*	GCA_000711215, Gp0047018, Ga0005617, HUN195	Paynter and Hungate (1968), Balch et al. (1979), Tanner and Wolfe (1988), Kuhner et al. (1991), Sprott and Beveridge (1993), Regensbogenova et al. (2004), and Seshadri et al. (2018)
*Methanobacterium* spp.								
*Methanobacterium bryantii* (DSM 863, ATCC 33272)	M.o.H., anaerobic sewage digester	H_2_ + CO_2_ [stimulatory – acetate, cysteine, and B-vitamins (most stimulating biotin, folate, cobalamin)]	PM	6.9–7.2, 37–39	ND	NA	GCA_002287175, Gp0322642, Ga0308562, NA	Bryant et al. (1971) and Balch et al. (1979)
*Methanobacterium formicicum* (NA)	BRM9, cow rumen	H_2_ + CO_2_, formate [required – yeast extract, rumen fluid]	PM	6.5–7.0, 39	2.6, RF30, H_2_/CO_2_ (80:20)	NA	GCA_000762265, Gp0007264, Ga0069308, NonHun80	Jarvis et al. (2000) and Kelly et al. (2014)
*Methanosarcina* spp.								
*Methanosarcina barkeri* (NA)	CM1, cow rumen	H_2_ + CO_2_, methanol, methylamine, trimethylamine, acetate [required – rumen fluid]	MC + S-layer	6.5, 39	5.4, RF30, H_2_/CO_2_ (80:20)	NA	GCA_001027005, Gp0007672, Ga0077912, NonHun91	Jarvis et al. (2000)
*Methanosarcina thermophila* (DSM 11855)	Ms97, sheep rumen	H_2_ + CO_2_, methanol, [required – yeast extract, rumen fluid]	MC + S-layer	6.5–6.8, 50	NA	NA	GCA_900116525, Gp0087973, Ga0007631, HUN272	Seshadri et al. (2018) and Zhou et al. (2021)
*Methanomassiliicoccaceae*								
*Methanomassiliicoccaceae* Group 12 (NA)	ISO4-H5, sheep rumen	H_2_ + methanol/mono−/di−/trimethylamine [required – yeast extract, acetate, formate, rumen fluid, coenzyme M]	Bi-CM	NR, 38–39	NA	NA	GCA_001560915, Gp0125684, Ga0114162, NonHun78	Jeyanathan (2010) and Li et al. (2016)
*Methanomassiliicoccaceae* Group 11 (NA)	ISO4-G1, sheep rumen	H_2_ + methanol/mono−/di−/trimethylamine [required – yeast extract, acetate, formate, rumen fluid, coenzyme M]	Bi-CM	NR, 38–39	NA	NA	GCA_001563305, Gp0139499, Ga0118695, NonHun77	Jeyanathan (2010), Kelly et al. (2016a,b)
Methanogenic archaeon (NA)	ISO4-G11, sheep rumen	H_2_ + methanol [required – yeast extract, acetate, formate, rumen fluid, coenzyme M]	Bi-CM	NR, 38–39	NA	NA	NA	Jeyanathan (2010)
Methanogenic archaeon (NA)	RumEn M1, cow rumen	H_2_ + trimethylamine [required – acetate, formate, rumen fluid]	Bi-CM	NR, 37	NA	NA	LJKK00000000, NA, NA, NA	Söllinger et al. (2016)
Methanogenic archaeon (NA)	RumEn M2, cow rumen	H_2_ + trimethylamine [required – acetate, formate, rumen fluid]	Bi-CM	NR, 37	NA	NA	LJKL00000000, NA, NA, NA	Söllinger et al. (2016)

NA, not available; ND, not determined; NR, not reported; PM, pseudomurein; RS, regularly structured; MC, methanochondroitin; Bi-CM, thin bi-layer cell membrane. BRN media, not specified (Rea et al., 2007); RF30 media, rumen fluid-containing BY medium (Jeyanathan, 2010); [potentially], genome contains gene homologs of adhesin-like protein.

#### 3.1.1. Methanobacteriales

Members of this order reduce CO_2_ with H_2_ and some use formate, CO, and secondary alcohols as reductants (Liu, 2010a); *Methanosphaera,* an exception, reduce methanol with H_2_ (Miller and Wolin, 1985). Their cell walls contain an archaeal-type peptidoglycan composed of N-acetyltalosaminuronic acid with β-1,3 glycosidic bonds and L-amino acid peptide crosslinks (König and Kandler, 1979; Sprott and Beveridge, 1993). Most members are mesophiles, and the respective genera occur in the ruminant digestive tract (Liu and Whitman, 2008; Liu, 2010a; Lyu and Liu, 2019).

##### 3.1.1.1. Methanobrevibacter (Mbb)

**Figure 3:**
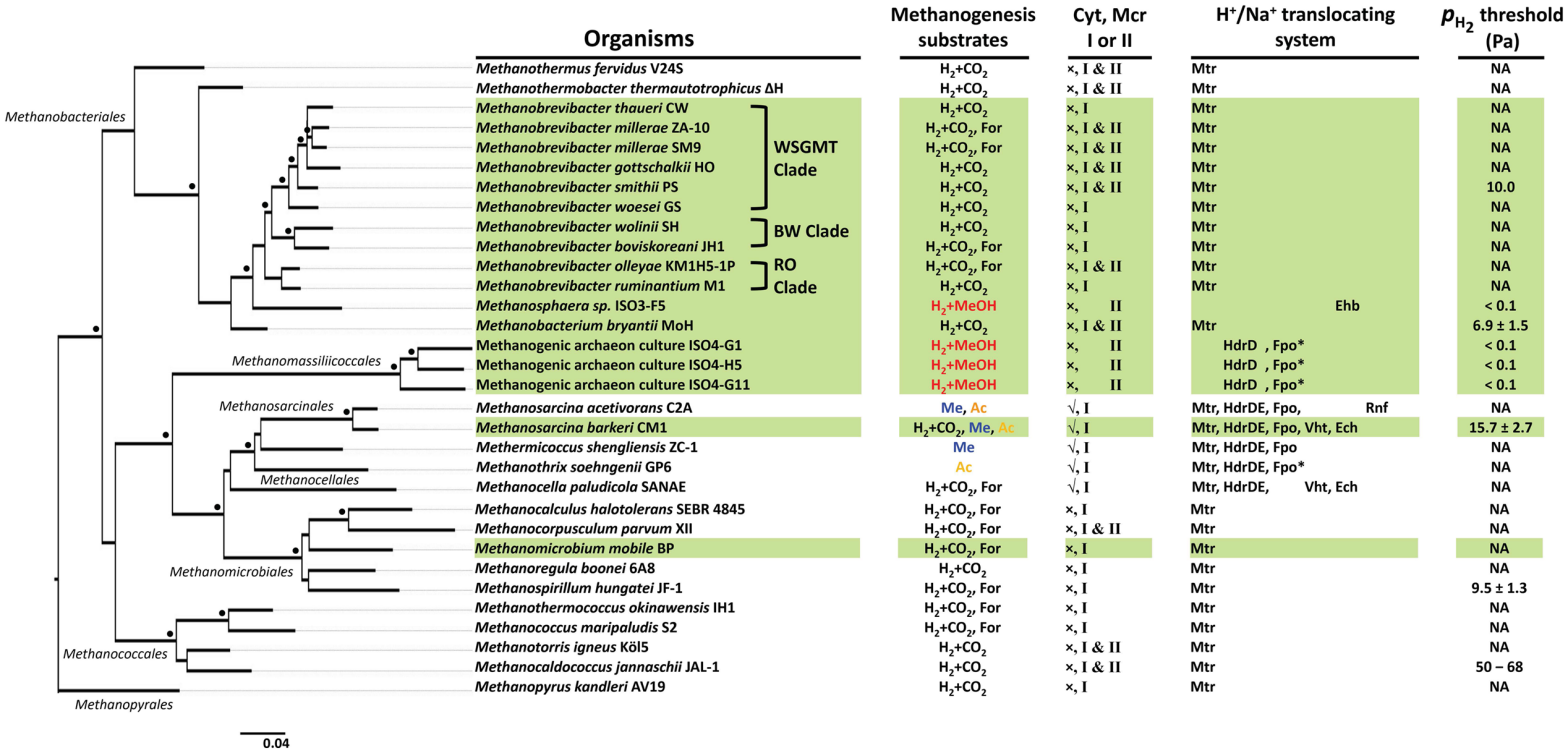
Phylogeny of rumen methanogenic archaea. A 16S ribosomal RNA (rRNA) gene sequence-based phylogenetic tree was constructed via a distance-based phylogeny inference algorithm at NGPhylogeny webserver (https://ngphylogeny.fr/) (Desper and Gascuel, 2002; Criscuolo and Gribaldo, 2010; Junier and Zdobnov, 2010; Katoh and Standley, 2013; Lefort et al., 2015; Lemoine et al., 2018) with *Desulfurococcus amylolyticus* Z-1312 16S rRNA gene sequence as an outgroup (not shown). Black dots at the branches, confidence values of ≥700 (out of 1,000 replicates). Scale bar, number of base substitutions per site. Mode of methanogenesis substrate use (as shown in Figure 5): black, CO_2_-reduction or formate-dependent; red, methyl-reduction with H_2_; blue, Methyl-dismutating; yellow, acetoclastic. Highlighted in green, rumen-associated methanogens. Sources of information in the H^+^/Na^+^ translocating system and $p_{H_{2}}$ threshold: (Deppenmeier, 2004; Thauer et al., 2008; Sakai et al., 2011; Ver Eecke et al., 2012; Welte and Deppenmeier, 2014; Kulkarni et al., 2018; Kröninger et al., 2019; Mand and Metcalf, 2019; Feldewert et al., 2020; Kurth et al., 2020, 2021; Downing et al., 2023). For, formate; MeOH, methanol; Me, methanol and mono-, di-, and trimethylamines; Ac, acetate; Mtr, methyl-tetrahydromethanopterin:coenzyme M methyltransferase; HdrDE, membrane-bound heterodisulfide reductase; Ech, energy-conserving hydrogenase; Ehb, a homolog of energy-conserving hydrogenase; Fpo, F_420_H_2_:phenazine oxidoreductase; Fpo*, Fpo in the absence of the F and O subunits; Vht, [NiFe] hydrogenase; Rnf, an equivalent of *Rhodobacter* nitrogen fixation complex; Mcr, methyl-coenzyme M reductase; cyt, cytochrome; √, cytochrome-containing; ×, cytochrome-non-containing.

These methanogens are major contributors in rumen methane production (Janssen and Kirs, 2008, Henderson et al., 2015). Approximately 74% of the 16S rRNA amplicons of rumen methanogens from rumen samples are affiliated with *Mbb. gottschalkii* and *Mbb. ruminantium* (Janssen and Kirs, 2008; Henderson et al., 2015). Thus far, only a few rumen *Methanobrevibacter* species have been isolated from the rumen (Table 1) and they form two phylogenetic clades, *smithii*-*gottschalkii*-*millerae*-*thaurei* (SGMT) and *ruminantium-olleyae* (RO) (Table 1 and Figure 3; King et al., 2011). These clades’ abundance and distribution vary over hosts and diets (St-Pierre et al., 2015), with generally one clade dominating over the other (Wright et al., 2007; Yeoman and White, 2014; Seedorf et al., 2015), and in only a few instances these exhibiting balanced abundances (Wright et al., 2007; St-Pierre et al., 2015). From a phylogenetic analysis that included *Mbb. woesei*, *Mbb. wolinii*, and *Mbb. boviskoreani* (Figure 3), we propose to expand the SGMT into the *woesei-smithii*-*gottschalkii*-*millerae*-*thaurei* (WSGMT) and form a new clade of *boviskoreani-wolinii* (BW), while retaining the RO clade (Figure 3).

**Figure 4:**
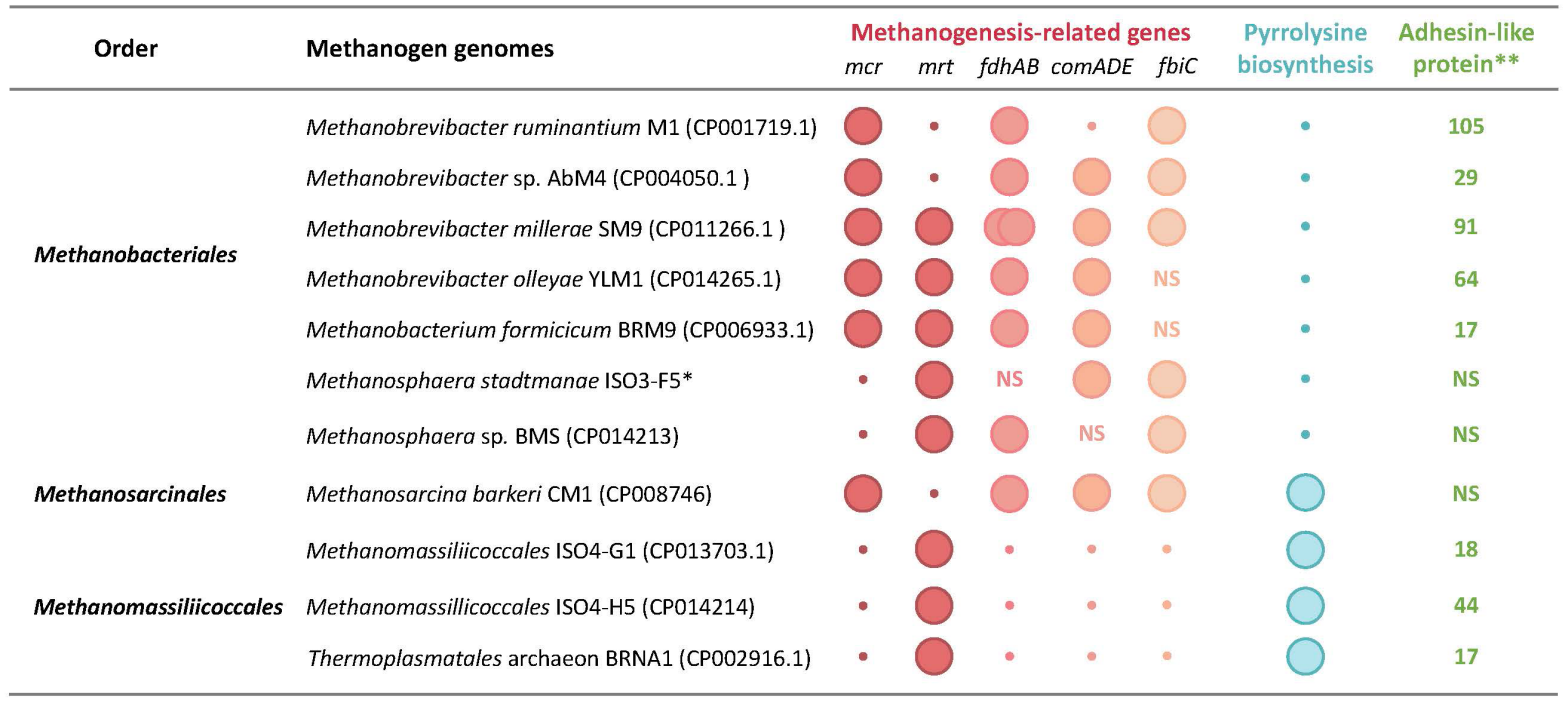
Unique genomic features of rumen methanogens. Genomic features: *mcr* and *mrt*, McrI and McrII isozymes of methyl-CoM reductase, respectively; *fdhAB,* two subunits of formate dehydrogenase as utilized for formate utilization*; comADE* and *fbiC,* CoM and F_420_ biosynthetic genes; *pylBC*D, genes encoding pyrrolysine biosynthesis enzymes; *pylT,* transfer RNA for pyrrolysine (tRNA^Pyl^); *pylS*, pyrrolysyl-tRNA synthetase (PylRS); *, total number of adhesin-like protein; NS, not specified. The data are from Jeyanathan (2010), Leahy et al. (2010, 2013), Kelly et al. (2014, 2016a,b,c), Lambie et al. (2015), Li (2016), and Li et al. (2016).

Within the WSGMT clade, the presence of *Mbb. smithii* in the rumen system is questionable (Janssen and Kirs, 2008; Table 1 and Supplementary Table S1), as it was originally isolated from a sewage digester (Balch et al., 1979) and others were isolated from human feces and large intestine (Miller and Wolin, 1981; Miller et al., 1982). Rare detection of *Mbb. smithii*-like organisms in rumen have been based on the 16S rRNA sequence analysis (Supplementary Table S1). *Methanobrevibacter* species can produce methane from CO_2_-reduction with H_2_ and formate. The genomes of rumen methanogens often lack essential biosynthetic genes, such as those for coenzyme M, perhaps due to gene loss from prototrophic ancestors (Figure 4, Section 3.3), and in the rumen, resulting auxotrophies are supported with supplements from other organisms, including other methanogens (Hazlewood and Dawson, 1977). These auxotrophies often make the laboratory cultivation of rumen *Methanobrevibacter* species quite tedious, since growth factor(s), such as coenzyme M, short-chain fatty acids, amino acids, acetate, and vitamins need to be provided (Bryant et al., 1971; Balch and Wolfe, 1976; Balch et al., 1979; Miller and Lin, 2002; Rea et al., 2007; Lee et al., 2013; Table 1). Branched-chain volatile fatty acids, especially 2-methylbutyrate and isovalerate, are used for amino acid synthesis of isoleucine and leucine, respectively (Whitman et al., 1982; Shieh et al., 1988). In some cases, because of the unknown type auxotrophies, supplementation with rumen fluid is necessary (Bryant et al., 1971; Balch and Wolfe, 1976; Balch et al., 1979; Miller and Lin, 2002; Rea et al., 2007; Lee et al., 2013). Certain *Methanobrevibacter* species express adhesin-like proteins that likely allow symbiosis with ciliates and other hydrogen producers (Figure 4; Ng et al., 2016; Patra et al., 2017).

##### 3.1.1.2. Methanosphaera (Msp)

*Msp. stadtmanae* ISO3-F5 and *Msp.* BMS are the sole rumen isolates of the *Methanosphaera* genus (Jeyanathan, 2010; Hoedt et al., 2018) and the former is closely related to the human fecal isolate *Msp. stadtmanae* MCB-3 (Miller and Wolin, 1985) with a 16S rRNA sequence similarity of 96% (Jeyanathan, 2010). *Methanosphaera* species are obligate H_2_-dependent methylotrophs (Miller and Wolin, 1985; Jeyanathan, 2010). The genome sequence of *Msp.* BMS but not *Msp. stadtmanae* ISO3-F5 is available (Jeyanathan, 2010; Hoedt et al., 2018). Rumen isolates require several growth factors, such as yeast extract, acetate, and fatty acids (Table 1). *Msp. stadtmanae* occurs in a free state as well as a symbiont of the rumen protozoa, *Eudiplodinium* and *Entodinium* (Tymensen et al., 2012; Xia et al., 2014).

##### 3.1.1.3. Methanobacterium (Mb)

*Mb. formicicum* BRM9 is the rumen representative of this genus, and this cow rumen isolate uses H_2_ + CO_2_ and formate for methanogenesis (Jarvis et al., 2000). It requires yeast extract and fatty acids for growth (Table 1).

#### 3.1.2. Methanomassiliicoccales

*Methanomassiliicoccales* order of the more recently recognized phyla of Candidatus Thermoplasmatota represent the second most abundant methanogen group after *Methanobrevibacter* in the rumen (Henderson et al., 2015). It is currently represented by five families, four genera, and one pure culture isolate, *Methanomassiliicoccus luminyensis* B10 of *Methanomassiliicoccaceae* family obtained from human feces (Dridi et al., 2012). *Methanomassiliicoccales* are mesophiles and mostly associated with animal gastrointestinal tracts (Dridi et al., 2012; Li et al., 2016; Söllinger et al., 2016; Kelly et al., 2016a,b; Cozannet et al., 2020). The strain B10 derives energy from H_2_-dependent methanogenesis from methylated compounds, such as methanol, methyl-, dimethyl-, and trimethylamine (Dridi et al., 2012; Li et al., 2016; Kelly et al., 2016a,b). Similar to Mycoplasma, which are cell wall deficient bacteria (Brown et al., 2018), *Methanomassiliicoccales* lack the archaeal S-layer cell wall and possess a bi-layer cell membrane (Dridi et al., 2012; Li et al., 2016), which in strain B10 contains unusual butane- and pentanetriol-based tetraether lipids (Becker et al., 2016).

There are reports on the enrichment of rumen *Methanomassiliicoccales*, and ISO4-H5, ISO4-G1, ISO4-G11, RumEn M1, and RumEn M2 are such examples (Kelly et al., 2016a,b; Li et al., 2016; Söllinger et al., 2016). These isolates rely exclusively on H_2_-dependent methyl-reducing methanogenesis for energy production (Kelly et al., 2016a,b; Li et al., 2016; Söllinger et al., 2016), and genome analysis suggests that ISO4-H5 and ISO4-G1 are coenzyme M auxotrophs (Li et al., 2016; Kelly et al., 2016a,b); the genome sequence of ISO4-G11 is not available (Jeyanathan, 2010) and that of RumEn M1 and RumEn M2 are incomplete (Söllinger et al., 2016). Further investigations on the physiology of these methanogens will require isolation in pure cultures.

#### 3.1.3. Methanomicrobiales

The species of this order representing eight families perform methanogenesis from CO_2_ with H_2_, formate, and secondary alcohol as electron sources (Zellner and Winter, 1987; Liu, 2010b). *Methanomicrobium mobile* BP, a bovine isolate that uses H_2_ + CO_2_ and formate (Paynter and Hungate, 1968) and belongs to the *Methanomicrobiaceae* family, is the only rumen representative of this order (Table 1). It constitutes only a small fraction of rumen methanogen population (Henderson et al., 2015) and forms symbioses with ciliates via an unknown mechanism (Regensbogenova et al., 2004). *Mm. mobile* has the most complex growth factor requirements among methanogens (Table 1); the nature of a factor that is called mobile element and could be provided from boiled cell extract of *Methanothermobacter thermautotrophicus* remains unknown (Tanner and Wolfe, 1988; Kuhner et al., 1991; Table 1).

#### 3.1.4. Methanosarcinales

The rumen representatives of this order are *Methanosarcina barkeri* CM1 and *Methanosarcina thermophila* Ms97 that belong to the *Methanosarcinaceae* family (Table 1), and like other *Methanosarcina*, they are metabolically versatile and can utilize H_2_ and CO_2_, methylated compounds, such as methanol, methylamines, and methanethiol, and acetate, for methane production (Table 1; Rospert et al., 1990; Reeve, 1992; Morgan et al., 1997; Reeve et al., 1997; Deppenmeier et al., 2002; Galagan et al., 2002; Maeder et al., 2006; Lambie et al., 2015). The rumen isolates require yeast extract and rumen fluids for growth (Table 1). While co-culture experiments show symbiotic interactions of a non-rumen isolate of *Ms. barkeri* with ruminal fungi and ciliates (Mountfort et al., 1982; Hillman et al., 1988; Ushida et al., 1997), no such information is available for a rumen *Methanosarcina* (Lambie et al., 2015).

#### 3.1.5. Methanotrichales

*Methanothrix* species are the sole members of this order (Garrity et al., 2011) and are known to obtain energy solely from acetoclastic methanogenesis (Lyu and Liu, 2019; Akinyemi et al., 2021), although their genomes suggest a capability of CO_2_-reduction with H_2_ and CO as electron sources (Smith and Ingram-Smith, 2007). A low abundance of 16S rRNA gene sequences representing *Methanothrix concilii* have been detected in rumen samples (Henderson et al., 2015).

### 3.2. Energy metabolism and physiology

For energy production, methanogens rely on methanogenesis, and based on the methanogenic substrates utilized, these archaea are divided into three groups (substrate, group name): hydrogen and formate as electron donor for CO_2_ reduction (hydrogenotrophic and formate-dependent, respectively); methyl-containing compounds and acetate as sources of both methyl group and electron source (methylotrophic and acetoclastic, respectively) (Wolfe, 1992). However, for the recently emphasized role of methanogens that remove H_2_ via methyl group reduction in the rumen, human gut, and many other ecological niches, the definition of hydrogenotrophic methanogenesis has been expanded to the following (hydrogenotrophic pathway, associated methanogens): CO_2_-reducing hydrogenotrophy (CO_2_-reducing hydrogenotrophs) and methyl-reducing hydrogenotrophy (methyl-reducing hydrogenotrophs) (Garcia et al., 2022; Bueno de Mesquita et al., 2023). Similarly, methanogenesis from CO_2_ with formate and secondary alcohols as reductants, where the electrons are recovered from the primary donor as F_420_H_2_ (Thauer et al., 2008; Yan and Ferry, 2018) could be called formate-dependent and secondary alcohol-dependent methanogenesis, respectively; for the former, CO_2_-reducing formatotrophic name has also been proposed (Garcia et al., 2022). In the following subsections, each of the methanogenesis pathways and the corresponding energy conservation strategies are described and linked to the rumen methanogens that employ them.

#### 3.2.1. CO_2_-reducing hydrogenotrophy and formate-dependent methanogenesis

**Figure 5:**
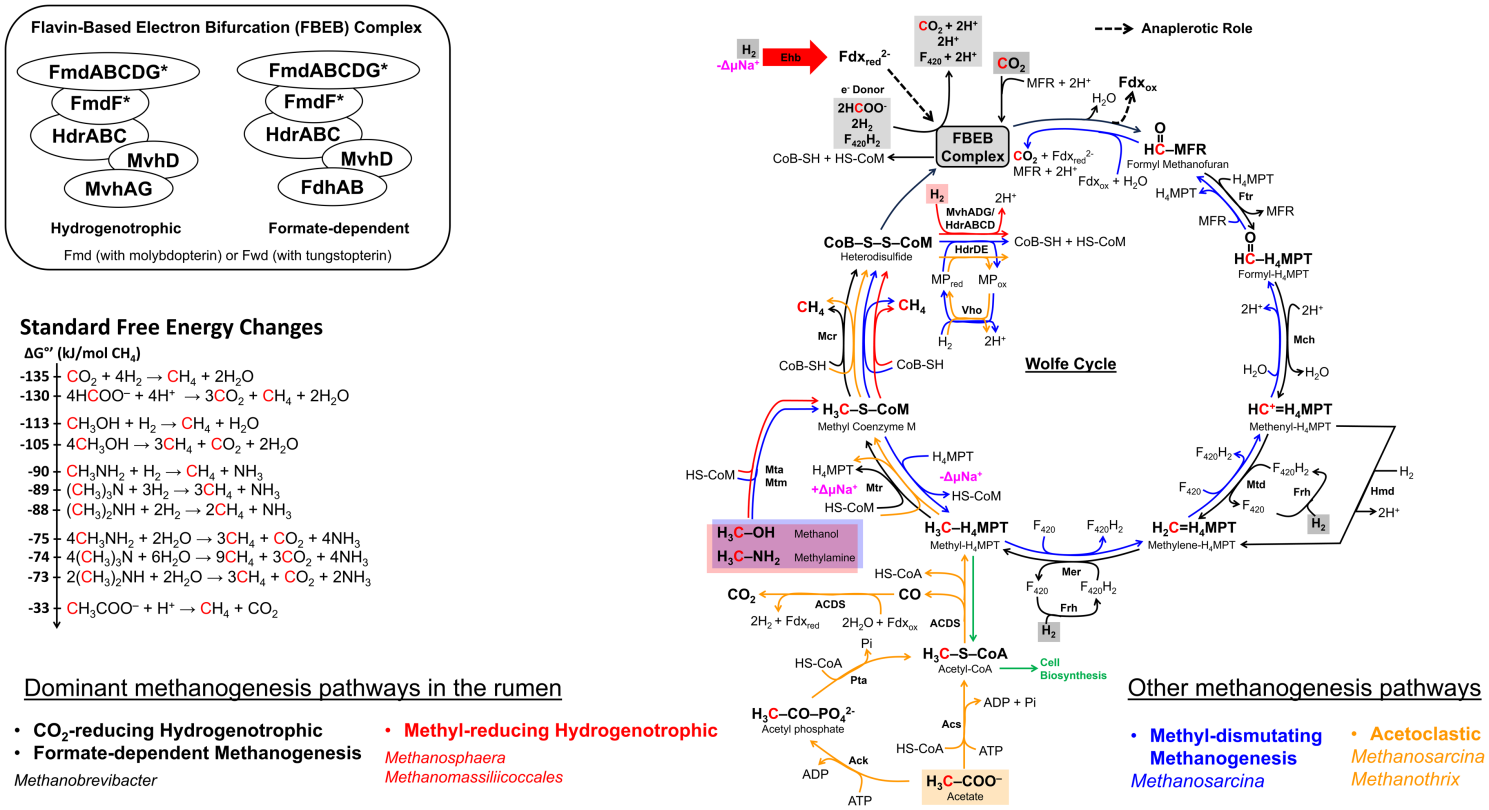
The methanogenesis cycle, energy conservation, and associated standard free energy changes. The cycle is first proposed by Rouvière and Wolfe (1988) and subsequent discovery in flavin-based electron bifurcation (FBEB) makes the cycle full circle (Thauer, 2012; Watanabe et al., 2021). Pathway arrow colors: black, CO_2_-reducing hydrogenotrophic or formate-dependent methanogenesis (Wood et al., 2003; Costa et al., 2010; Thauer, 2012); red, blue, and orange, methyl-reducing hydrogenotrophic, methyl-dismutating, and acetoclastic, respectively (Welander and Metcalf, 2005; Kurth et al., 2020). The FBEB complex which consists of Mvh-Hdr-Fmd/Fwd or Fdh-Hdr-Fmd/Fwd is proposed to be a common structure in CO_2_-reducing or formate-dependent methanogens without cytochromes (Watanabe et al., 2021). Values for standard free energy changes are taken from Liu and Whitman (2008) or calculated from Thauer et al. (1977). Fdx_red_, reduced ferredoxin; Fdx_ox_, oxidized ferredoxin; MFR, methanofuran; H_4_MPT, tetrahydromethanopterin; MP, methanophenazine; CoB-SH or CoB, coenzyme B; CoM-SH or CoM, coenzyme M; Fmd, molydopterin containing formyl-MFR dehydrogenase; Ftr, formyl-MFR:H_4_MPT formyltransferase; Mch, methenyl-H_4_MPT cyclohydrolase; Mtd, methylene-H_4_MPT dehydrogenase; Hmd, H_2_-dependent methylene-H_4_MPT dehydrogenase; Mer, methylene-H_4_MPT reductase; Mtr, methyl-H_4_MPT:CoM methyltransferase; Mcr, methyl-CoM reductase; Hdr, electron bifurcating hydrogenase-heterodisulfide reductase complex; HdrABC, soluble heterodisulfide reductase; HdrDE, membrane-bound heterodisulfide reductase; Mvh, non-F_420_-reducing hydrogenase; Frh, F_420_-reducing hydrogenase; Mta, methylcobamide:CoM methyltransferase; Mtm, monomethylamine methyltransferase; Acs, acetyl-CoA synthase; Ack, acetate kinase; Pta, phosphotransacetylase; ACDS, acetyl-CoA decarbonylase/synthase; Fdh, formate dehydrogenase; Eha, a homolog of energy-conserving hydrogenase; Vho, methanophenazine-dependent hydrogenase; ΔμNa^+^, electrochemical sodium ion potential.

CO_2_-reducing hydrogenotrophy (Figure 5) is one of the most ancient respiratory metabolisms on Earth (Leigh, 2002; Teske et al., 2003). Here, CO_2_ is first reduced to a formyl group, which is dehydrated to methenyl and then sequentially reduced to methylene and methyl groups and finally, to methane (Figure 5); three coenzymes, methanofuran (MFR), tetrahydromethanopterin (H_4_MPT), and coenzyme M (CoM-SH or CoM) act as carriers for the carbon units at four oxidation states (+4, +2, 0, and − 2) (Wolfe, 1992). Reduced coenzyme F_420_ (F_420_H_2_), generated by an F_420_-reducing [NiFe]-hydrogenase (Frh) with H_2_, serves as a direct electron donor for the reduction of methenyl and methylene forms. Coenzyme B (CoB-SH or CoB) helps to reduce the methyl group of CH_3_-S-CoM to CH_4_, and this process generates heterodisulfide of CoM and CoB (CoM-S-S-CoB) (Wolfe, 1992; Thauer et al., 2010; Thauer, 2012).

In CO_2_-hydrogenotrophic methanogens without cytochromes such as *Methanobrevibacter*, the only site for energy conservation is the sodium translocating membrane-associated methyl-H_4_MPT:CoM methyltransferase complex composed of MtrA–H subunits (Figure 5; Thauer et al., 2008) and heterodisulfide reduction occurs as follows. A cytoplasmic complex composed of heterodisulfide reductase (HdrABC), non-F_420_-reducing hydrogenase (MvhADG), and formyl-methanofuran dehydrogenase (FmdABCDFG or FwdABCDFG) retrieves electrons from H_2_ (E^o^’, −420 mV), and bifurcates these using the FAD unit of HdrA to provide high potential electrons for the reduction of CoM-S-S-CoB (E^o^’, −140 mV) at HdrB and low potential electrons for formyl-MFR synthesis from CO_2_ (E^o^’, −500 mV) (Costa et al., 2010; Yan and Ferry, 2018; Watanabe et al., 2021; Figure 5); Fmd and Fwd are molybdo- and tungsto-pterin carrying isoenzymes of formyl-methanofuran dehydrogenase, respectively (Schmitz et al., 1992). For methanogenesis with formate, as discussed below, the MvhADG unit is replaced with F_420_-reducing formate dehydrogenase (FdhAB) that can obtain electrons from either formate using FdhA or F_420_H_2_ via FdhB (Costa et al., 2010; Watanabe et al., 2021). The direct electronic coupling of the first (formyl-methanofuran synthesis) and last (CoM-S-S-CoB reduction) steps of methanogenesis generates a cyclic system that has been called the Wolfe Cycle, named after Ralph Wolfe (Rouvière and Wolfe, 1988; Thauer, 2012; Figure 5). When the electron bifurcation falls short, an energy-converting hydrogenase (Eha) provides low potential electrons for formyl-MFR synthesis via a ferredoxin, serving an anaplerotic function (Figure 5; Lie et al., 2012).

For methanogens with cytochromes, electrons derived from H_2_ by the action of a membrane-bound and proton pumping VhoAGC hydrogenase complex are channeled to HdrDE for CoM-S-S-CoB reduction, and the low-potential Fdx_red_ that are needed for formyl-MFR synthesis are generated via another membrane-bound hydrogenase complex (EchA-F) that is aided by a proton-motive force (Thauer et al., 2008). Thus, a cytochrome carrying CO_2_-hydrogenotroph such as *Methanosarcina* has two sites of energy conservation, Mtr and VhoAGC (Thauer et al., 2008).

Under standard conditions, the hydrogenotrophic mode is the most exergonic of all methanogenesis systems (ΔG°′ = −135 kJ/mol CH_4_) (Figure 5). However, under the rumen conditions (hydrogen partial pressure or $p_{H_{2}}$ ), 162 Pa (Barry et al., 1977; Ungerfeld and Kohn, 2006), the prevailing ΔG’ value of the hydrogenotrophic methane formation reaction is only −67.4 kJ/mol CH_4_ (Ungerfeld and Kohn, 2006) and yet, *Mbb. ruminantium* and *Mbb. gottschalkii* together represent as high as 74% of the total archaeal community (Henderson et al., 2015). It has been suggested that the flavin-dependent bifurcation system producing a low potential reduced Fdx pool is a key tool for a methanogen living under low $p_{H_{2}}$ (Yan and Ferry, 2018).

The genome of *Mbb. ruminantium* strain M1 carries all the genes necessary for methane production from H_2_ and CO_2_ (Leahy et al., 2010). It also carries a locus with genes for a formate transporter (*fdhC*, *mru_0332*), a formate dehydrogenase (*fdhAB, mru_0333* and *mru_0334*), and genes encoding molybdopterin-guanine dinucleotide biosynthesis (*moa, mru_0335* and *mru_0336*), enabling the organism to transport and oxidize formate; formate dehydrogenase contains a molybdopterin cofactor (May et al., 1986; Reeve, 1993).

There are indications that various groups of *Methanobrevibacter* use two different isoenzymes of methyl-coenzyme M reductase (Mcr) which catalyzes methane production from CH_3_-S-CoM (Figure 5). This difference has a major implication for their hydrogen metabolism (Reeve, 1992; Bonacker et al., 1993). The Mcr isoenzymes, Mcr I and McrII, encoded by the *mcr* and *mrt* genes, respectively, are considered physiologically adapted to function at low and high $p_{H_{2}}$ values (Rospert et al., 1990; Reeve et al., 1997). *Mbb. ruminantium* M1, a RO group organism, with *mcrBCDGA* genes encodes only McrI (Leahy et al., 2010). Of the rumen isolates from the WSGMT group, only for *Mbb. millerae* SM9’s complete genome sequence is available, and it carries both *mcr* and *mrt* genes (Kelly et al., 2016c).

#### 3.2.2. Methyl-reducing hydrogenotrophy

In this process, the methyl group from methylated compounds, such as methanol, methylamine, and methanethiol are transferred to CoM to form methyl-CoM which is then reduced to methane by Mcr (Figure 5), and H_2_ serves as the primary electron source for CoM-S-S-CoB reduction (Figure 5; Keltjens and Vogels, 1993). In the rumen, only methanol and methylamines (either mono-, di-, or trimethylamines), but not methanethiol, are available for this metabolism (Miller and Wolin, 1985; Fricke et al., 2006; Jeyanathan, 2010; Henderson et al., 2015; Kelly et al., 2016a,b; Li et al., 2016).

The ΔG^o^’ value for this process under standard conditions is −113 kJ/mol CH_4_, making it the second most exergonic methanogenesis process (Figure 5). The ΔG’ values under rumen conditions, however, have not been reported and would be variable as methanol and methylamine concentrations are dependent on the host diet, and $p_{H_{2}}$ also varies (Henderson et al., 2015). The methyl-hydrogenotrophs constitute about 16% of the rumen archaeal community and along with CO_2_-hydrogenotrophs and formate-utilizers, these organisms cover close to 90% of the methanogens in this habitat (Henderson et al., 2015). These methyl-hydrogenotrophic rumen methanogens belong to *Methanosphaera* sp. and two *Methanomassiliicoccales*-affiliated groups (Miller and Wolin, 1985; Fricke et al., 2006; Jeyanathan, 2010; Henderson et al., 2015; Li et al., 2016; Kelly et al., 2016a,b); *Methanosarcina,* which also can perform methyl-hydrogenotrophy (Mukhopadhyay et al., 1993) are rarely encountered in the rumen (Henderson et al., 2015).

The metabolic potential of *Msp* genus was inferred from the genome sequence analyses of a human fecal isolate, *Msp*. *stadtmanae* MCB-3 (Fricke et al., 2006), and that of rumen strain BMS (Hoedt et al., 2018). Genome analysis of MCB-3 showed that it lacks the genes for the biosynthesis of molybdopterin, an essential prosthetic group of formylmethanofuran dehydrogenase (Fmd), making the organism incapable of activating CO_2_ to the formyl stage and performing CO_2_-hydrogenotrophic methanogenesis (Fricke et al., 2006; Figure 5). The organism also lacks the genes for the synthesis of acetyl-CoA decarbonylase/synthase complex, which explains the requirement of acetate for its growth and its inability to utilize acetate for methanogenesis (Miller and Wolin, 1985). All these phenotypes have been observed in the rumen strains ISO3-F5 and BMS (Jeyanathan, 2010; Hoedt et al., 2018) which relies solely on H_2_ and methanol for methane production (Jeyanathan, 2010; Hoedt et al., 2018; Figure 5). Based on the presence of *mrt* and absence of *mcr* in the genome of MCB-3, it is inferred that the ISO3-F5 strain uses McrII (Fricke et al., 2006; Jeyanathan, 2010), which likely operates at high $p_{H_{2}}$ values (Rospert et al., 1990). The growth of BMS strain was also greatly enhanced at high $p_{H_{2}}$ , suggesting a dependence on McrII as well (Figure 5; Hoedt et al., 2018). The energy conservation system in *Methanosphaera* relies on the generation of reduced Fdx by the electron bifurcating HdrABC/MvhADG complex, and the free energy of the reduced Fdx is used for sodium ion translocation via membrane-bound energy-conserving hydrogenase (Ehb) complex (Fricke et al., 2006; Thauer et al., 2008; Yan and Ferry, 2018).

The genomes of *Methanomassiliicoccales* strains ISO4-H5, ISO4-G1, RumEn M1, and RumEn M2 (Jeyanathan, 2010; Li et al., 2016; Söllinger et al., 2016; Kelly et al., 2016a,b) lack the genes for many of the enzymes that are required to reduce CO_2_ to the methyl stage or to oxidize the methyl group of methyl-CoM to CO_2_ that could provide reductants for methyl-coenzyme M reduction (Lang et al., 2015; Li et al., 2016; Söllinger et al., 2016; Kelly et al., 2016a,b; Figure 5). Consequently, members of the *Methanomassiliicoccales* order are restricted to methyl-hydrogenotrophy; as mentioned above, for *Methanosphaera* species, such a restriction is due to a narrower reason, an inability to biosynthesize the molybdopterin cofactor for Fmd.

Above-mentioned rumen methanogens of the *Methanomassiliicoccales* order contain a F_420_H_2_:MP oxidoreductase-like (Fpo-like) complex and this could translocate protons for energy conservation (Lang et al., 2015; Li et al., 2016; Söllinger et al., 2016; Kelly et al., 2016a,b). However, they lack the genes for coenzyme F_420_, cytochrome, MP biosynthesis, and FpoF and FpoO subunits (Li et al., 2016; Söllinger et al., 2016; Kelly et al., 2016a,b) which in a Fpo complex of *Methanosarcina* species interact with F_420_H_2_ and MP, respectively (Welte and Deppenmeier, 2011); RumEn M2 strain also lacks the FpoA subunit (Söllinger et al., 2016). These genomes encode MvhADG, HdrABC, and HdrD but not HdrE (Lang et al., 2015; Li et al., 2016; Kelly et al., 2016a,b). Thus, it is possible that in rumen representatives of the *Methanomassiliicoccales* order, the Fpo-like complex couples the oxidation of bifurcation-derived reduced Fdx to the formation of a proton gradient (Kröninger et al., 2019). *Methanomassiliicoccales* carry *mrt* genes and lack the *mcr* system (Li et al., 2016; Kelly et al., 2016a,b), and therefore, utilize McrII which is known to operate at higher $p_{H_{2}}$ values (Rospert et al., 1990; Reeve, 1992; Reeve et al., 1997); as mentioned above, a similar situation exists with the *Methanosphaera* spp.

#### 3.2.3. Methylotrophic (or methyl-dismutating) and acetoclastic methanogenesis

Recently the term methyl-dismutating methanogenesis has been proposed as an alternate for methylotrophic methanogenesis (Garcia, Gribaldo et al., 2022). As the former embodies the mechanism of the process (Wolfe, 1992), we use this term for the rest of the narrative. Methyl-dismutating and acetoclastic methanogenesis are not significant processes in the rumen and the associated methanogens, *Methanosarcina* and *Methanothrix* species, are rarely encountered in this system (Hungate et al., 1970; Janssen and Kirs, 2008; Henderson et al., 2015; Seshadri et al., 2018); *Ms. barkeri* CM1 and *Ms. thermophila* Ms97 are the two rumen isolates (Lambie et al., 2015; Zhou et al., 2021). *Methanosarcina* species carry the *mcr* system and lack *mrt* genes (Deppenmeier et al., 2002; Galagan et al., 2002; Maeder et al., 2006; Lambie et al., 2015), hence these methanogens employ McrI that has been postulated to operate under low $p_{H_{2}}$ conditions (Rospert et al., 1990; Reeve, 1992; Morgan et al., 1997; Reeve et al., 1997). *Methanothrix* spp. carry the *mrt* system that generates Mcr II (Barber et al., 2011; Zhu et al., 2012).

In methyl-dismutating methanogenesis, one-fourth of the available methyl groups are oxidized, generating F_420_H_2_ and reduced Fdx which in turn allows the reduction of the rest of the methyl groups to methane, (4CH_3_X + H_2_O → 3CH_4_ + CO_2_; X = −OH, −NH_3_, and −SH) (Figure 5; Deppenmeier et al., 1996; Deppenmeier, 2004; Buan and Metcalf, 2010; Yan and Ferry, 2018). Following are the ΔG^o^’ values (kJ/mol CH_4_) for this process with the indicated substrates: −105 (methanol), −74 (trimethylamine), and − 49 (dimethylsulfide) [Figure 5; see reference (Liu and Whitman, 2008) for a comprehensive list]. The electrons from reduced Fdx, originating from the oxidation of formyl-MFR, are bifurcated to reduce CoM-S-S-CoB and to generate F_420_H_2_ (Deppenmeier et al., 1996; Deppenmeier, 2004; Buan and Metcalf, 2010; Yan and Ferry, 2018); F_420_H_2_ is also generated from the oxidation of methyl- and methylene-H_4_SPT (H_4_SPT, tetrahydrosarcinapterin, a variation of H_4_MPT). Then a FpoA-O complex couples the oxidation of F_420_H_2_ to proton translocation and also provides additional reductants for CoM-S-S-CoB reduction via methanophenazine (MP) and HdrDE (Deppenmeier et al., 1996; Deppenmeier, 2004; Buan and Metcalf, 2010; Yan and Ferry, 2018). Additional energy is generated by a Frh-based H_2_ cycling system that retrieves electrons from F_420_H_2_ via Frh and produces H_2_ and H^+^ gradient; the internally produced H_2_ diffuses out and is oxidized at the extra cytoplasmic location via VhtAGC to generate electrons that are transported to HdrDE via MP for heterodisulfide reduction (Kulkarni et al., 2018; Mand and Metcalf, 2019).

Of all types of methanogenesis, the acetoclastic mode has the least negative ΔG^o^’ value (−33 kJ/mol CH_4_) (Figure 5). Here, the methyl group of acetate is transferred to H_4_SPT for further processing, generating methane, CoM-S-S-CoB, and a Na^+^-motive force, and the oxidation of the carboxyl group provides reduced Fdx (Yan and Ferry, 2018). The reduced Fdx is utilized by the Rnf complex (equivalent of *Rhodobacter* nitrogen fixation complex) in two ways: first, Fdx^2−^ is oxidized employing cytochrome and the above-described MP- and HdrDE-mediated steps causing proton translocation and CoM-S-S-CoB reduction (Yan and Ferry, 2018; Mand and Metcalf, 2019); second, two Fdx^2−^ are processed with two oxidized F_420_, promoting sodium ion translocation and the resulting F_420_H_2_ are used by HdrA2B2C2 for the reduction of CoM-S-S-CoB and the production of Fdx^2−^ (Yan et al., 2017; Buckel and Thauer, 2018; Yan and Ferry, 2018). In both cases, a Na^+^/H^+^ antiporter adjusts the respective gradients for optimal ATP synthesis (Deppenmeier et al., 2002; Galagan et al., 2002; Maeder et al., 2006; Lambie et al., 2015; Yan and Ferry, 2018). A H_2_ cycling system similar to that described above for methyl-dismutating methanogenesis but with electrons derived from Fdx^2−^ via Ech, provides an additional avenue for energy production (Barber et al., 2011; Zhu et al., 2012; Kulkarni et al., 2018; Mand and Metcalf, 2019).

#### 3.2.4. Sources of methanogenesis substrates in rumen

The source of H_2_, formate, and acetate is predominantly carbohydrate fermentation as detailed above (Figure 2). Methanol is generated from de-esterification of methoxylated form of pectin, which is a polysaccharide component of the plant cell wall composed of alpha-1,4-galacturonic acid (Mitchell et al., 1979; Patterson and Hespell, 1979; Pol and Demeyer, 1988). This reaction is catalyzed by pectinase produced by *Butyrivibrio*, *Prevotella*, *Bacteroides*, *Ruminococcu*s, and *Fibrobacter* species (Comtet-Marre et al., 2017; Sollinger et al., 2018; Kelly et al., 2019); fungi, protozoa or associated bacteria also hydrolyze pectin (Wright, 1960). Degradation of choline and betaine, that are present in the feed (Mitchell et al., 1979; Patterson and Hespell, 1979; Pol and Demeyer, 1988) by choline-TMA lyase and betaine reductase, respectively, provides trimethylamine (TMA) (Craciun and Balskus, 2012; Rath et al., 2019). In the rumen, the choline-TMA lyase gene occurs in *Desulfovibrio*, *Clostridia*, *Streptococcus*, *Klebsiella*, and *Proteus* species (Craciun and Balskus, 2012) and betaine reductase is likely provided by *Eubacterium*, *Clostridium*, and various members of the Firmicutes (Naumann et al., 1983; Hormann and Andreesen, 1989; Rath et al., 2019). A recent study has provided the following values for the methyl-group containing substrate concentrations (μM) in bovine rumen fluid (Bica et al., 2022): methanol, 23–26; methylamine, 12–16; dimethylamine, 1.8–2.1; and trimethylamine, 1.6–2; the values were not significantly different across different diets. An earlier study in cattle and sheep rumens reported that the concentration of methylamine increases steadily during the 6–8 h period post-feeding and then decreases rapidly (Hill and Mangan, 1964). After an additional 5 h, methylamine was absent from the rumen and this status remained for a 24 h period that followed (Hill and Mangan, 1964). These data are consistent with a rapid utilization of methyl-group containing substrates by the methyl-hydrogenotrophs under the high $p_{H_{2}}$ condition following feeding (Sollinger et al., 2018).

### 3.3. Metabolic inferences from genome sequences

Identification of several gastrointestinal tract (GIT)- and rumen-associated microbes with reduced genome sizes that are smaller than that of the same species from non-host-associated niches suggest that nutrient-abundant nature of animal digestive tracts have facilitated genome streamlining events in these organisms (Walter and Ley, 2011; Söllinger et al., 2016). In some cases, GIT and rumen microorganisms gained additional genes (Leahy et al., 2013; Kelly et al., 2016c; Söllinger et al., 2016). For example, *Mbb. smithii* PS, a human gut-associated *Methanobrevibacter* species, can be distinguished from the rumen-associated *Methanobrevibacter* sp. Abm4 based on the presence of the *mtaABC* operon encoding methanol:cobalamin methyltransferase genes in the latter (Leahy et al., 2013). This is a surprise as *Methanobrevibacter* species are not known to utilize methanol (Boone et al., 1993) and the roles of *mtaABC* in strain Abm4 are unclear (Leahy et al., 2010, 2013). If these genes indeed allow H_2_-dependent methanogenesis from methanol in Abm4 similar to *Methanosphaera* and *Methanomassiliicoccales* or only on methanol as seen in *Methanosarcina*, these capabilities will introduce a major change in the concept of rumen methanogenesis. Remarkably, a comparison of genomes of rumen methanogens with those of closely related species originating from amoeba-associated and freshwater isolates has revealed higher metabolic versatility in the rumen methanogens (Kelly et al., 2014; Lambie et al., 2015).

Currently, for rumen methanogens at least 15 complete assembled genome sequences are available in public repositories (Supplementary Table S2), and these include that of *Mbb. boviskoreani* JH1 (Lee et al., 2013), *Methanoculleus bourgensis* KOR-2 (Battumur et al., 2019), and *Methanomassiliicoccales* RuMen M1 and M2 (Söllinger et al., 2016). The number increases further if those submitted as drafts or scaffolds are considered (Chen et al., 2023). Some of the genomes have been reported with corresponding publications (Jeyanathan, 2010; Leahy et al., 2010, 2013; Lee et al., 2013; Kelly et al., 2014, 2016a,b,c; Lambie et al., 2015; Li, 2016; Li et al., 2016; Söllinger et al., 2016; Battumur et al., 2019) and several, such as that for the *Thermoplasmatales* BRNA1 genome, have been deposited to the GenBank (accession number, CP002916) and not yet been reported in a publication.

Analyses of the methanogen genomes pinpoint specific gene markers that can be used to infer their metabolic capabilities. These markers include methanogenesis-related and cofactor biosynthesis genes (Leahy et al., 2010; Roehe et al., 2016; Sollinger et al., 2018; López-García et al., 2022; Figures 4, 5). Genes *fmdB* and *mtrA* that encode formylmethanofuran dehydrogenase subunit B and methyl-H_4_MPT:HS-CoM methyltransferase subunit A, respectively, for example, are effective markers for CO_2_-reducing hydrogenotrophs, whereas for methyl-reducing hydrogenotrophs, such as *Methanosphaera* and *Methanomassiliicoccales*, the markers are methanol- and methylamine-specific methyltransferase genes, *mtaB* and *mt*MA, respectively (Sollinger et al., 2018); *mt*MA represents a combination of mono-, di- and trimethylamine methyltransferase genes. An alignment of the sequences of the following seven core methanogenesis proteins extracted from whole genome sequences has been used in a taxonomic characterization of various methanogens from diverse ecological niches: four subunits of methyl-H_4_MPT:HS-CoM methyltransferase (MtrB, -C, -D, and -E); F_420_-dependent methylene tetrahydromethanopterin dehydrogenase, Mtd (Mukhopadhyay et al., 1995); coenzyme M biosynthesis enzyme, ComD (Graupner et al., 2000); and FO synthase subunit 1, CofG (Choi et al., 2002; Graham et al., 2003; Anderson et al., 2009) where FO is a core unit of coenzyme F_420_ (Eirich et al., 1979).

## 4. Adaptation of methanogens to the rumen ecosystems

Even the limited amount of data that are available for the relevant metabolic and genome characteristics clearly show evidence for the evolutionary developments that are specific to rumen methanogens as a member of a rumen microbial consortium. In the following sections, methanogen colonization and adaptation processes in the rumen are summarized.

### 4.1. Colonization of methanogens in the rumen and factors influencing rumen methanogen community composition

Calves are born with undeveloped rumens and function as monogastric animals. This development stage is also called the pre-ruminant phase (Church, 1988; Davis and Drackley, 1998). The reflective closure of the reticular groove bypasses the rumen and directs the feed, mostly milk or milk replacer, directly to the abomasum and then to small and large intestines (Van Soest, 1994). The rumen is established through three sequential steps, namely the development of rumen anatomy, fermentation capacity and function, and microbial colonization (Yáñez-Ruiz et al., 2015). This development occurs within the first several weeks or months of a calf’s life with a fully mature rumen forming following a major diet transition from colostrum in neonatal, and milk and a concentrate/grain-based feed for pre-weaned calves to solid feed in post-weaned calves.

Consumption of solid feed such as roughage or grains stimulates the development of rumen papillae for nutrient absorption, muscular structure for rumination, expansion of rumen capacity, and production of saliva (Tamate et al., 1962, Stobo et al., 1966; Lane and Jesse, 1997; Baldwin et al., 2004). In concert with these anatomical and feed changes, the rumen microbial community develops. Initial microbial colonization in the rumen occurs immediately after birth by diverse aerobes and facultative anaerobes (Fonty et al., 1987; Li et al., 2012; Jami et al., 2013). Several studies suggested that microbial colonization in the rumen may occur *in utero* between 5 and 7 months gestation or even much earlier such as at the end of the first trimester, although the mechanism of this transfer from mother to fetus is unclear (Guzman et al., 2020; Husso et al., 2021; Zhu et al., 2021; Amat et al., 2022).

These early occupants consume O_2_, and thus, provide an anoxic environment for obligate anaerobes that colonize by the second day of life (Fonty et al., 1987). Intriguingly, a study with euthanized Holstein bull calves detected a typical rumen microbial community comprised of methanogens, fibrolytic bacteria, and *Geobacter* spp. belonging to Proteobacteria phylum in the rumen fluid of dairy calves 20 min after their birth, suggesting that these microbes present in the GIT right after birth and long before the introduction of solid feed (Guzman et al., 2015). This finding is somewhat surprising given that these neonatal calves solely depend on colostrum and suckle milk for energy, and here, the rumen is bypassed. Thus, these observations are raising the question about the roles of these early microbial communities in the under-developed rumen.

Most studies of methanogen community in fully developed rumens point to the major abundance of CO_2_-reducing hydrogenotrophic methanogens (Janssen and Kirs, 2008). The information on methanogen community composition in pre-ruminants is scarce. *Methanomicrobium mobile*, *Methanococcus voltae*, and *Methanobrevibacter* sp., which are capable of utilizing H_2_ and formate, have been found in neonatal calves (Guzman et al., 2015). However, hydrogen is not considered to be the most prevalent electron source for methanogenesis at this stage. Instead, methanol and methylamine are used for methanogenesis in young animals, and species from *Methanosarcinales* order have been found to occur primarily in young and developing calves (Friedman et al., 2017).

This selection could be due to the presence of other hydrogen utilizers such as acetogens and sulfate reducers, which outcompete methanogens (Fonty et al., 1987, Morvan et al., 1994; Fonty et al., 2007). A study with gnotobiotically-reared lambs that were inoculated with functional methanogen-free rumen microbiota and then placed on solid feed has demonstrated that it is possible to establish a rumen system with hydrogenotrophic acetogens and sulfate-reducing bacteria as the main hydrogen sink (Fonty et al., 2007); this system persisted for 12 months after the initiation. It is noteworthy that the composition of the rumen methanogen community early in a calf’s life is also determined by the route of delivery and a lower abundance of methanogens is seen in vaginally delivered animals (Furman et al., 2020).

In addition to animal development stage, rumen microbial composition is influenced by factors such as host genetics and diets. Host genetics play roles in shaping the rumen microbiome and determining the efficiency of energy harvest from feed and extent of methane emission (Carberry et al., 2012; Jami et al., 2014; Kittelmann et al., 2014; McCann et al., 2014; Wallace et al., 2015; Roehe et al., 2016; Sasson et al., 2017; Difford et al., 2018; Zhang et al., 2020; Martínez-Álvaro et al., 2022). A link of the host genetics to the selection of twenty heritable microbes belonging to exclusively Bacteroidetes and Firmicutes phyla has been established (Sasson et al., 2017). However, the mechanisms underlying this observation remain to be clearly defined.

Of all factors influencing microbial community, diet composition and its physical characteristics such as particle size are considered as main drivers (Li et al., 2009; Henderson et al., 2015). A fiber-rich diet containing structural carbohydrates and large particles enriches fiber-degraders such as *Fibrobacter succinogenes*, *Ruminococcus flavifaciens*, and *Ruminococcus albus* (Johnson and Johnson, 1995). This type of diet also decreases feed digestion rate due to the presence of cell wall components that are less rapidly degraded than a starch-based diet, hence reducing feed passage rate and resulting in relatively higher methane emission (Janssen, 2010). Non-structural carbohydrate-rich diets, such as grains, concentrates, and readily fermented and small particle feed, shift the microbial community to one with *Butyrivibrio* spp. and *Succinivibrionaceae* as predominant members, increasing feed digestion and passage rate and resulting in lower methane emission (Tajima et al., 2001a; Luton et al., 2002; Tatsuoka et al., 2004; Friedrich, 2005; Janssen and Kirs, 2008; King et al., 2011; Henderson et al., 2015). Supplementary Table S3 summarized data on the methanogen communities in cattle fed various diets.

### 4.2. Leveraging auxotrophy in a nutrient-rich environment

The rumen is rich in nutrients and metabolites that are generated from the degradation of plant materials and microbial activities. Additionally, internal rumen environment is dynamic, due to the constant efflux of feed, ruminal passage rate, and nutrients absorption by the animals (Saleem et al., 2012; Ungerfeld, 2020; Malheiros et al., 2021; Bica et al., 2022). Such features encourage members of an ecosystem to interact and provide a fertile ground for horizontal gene transfer or native gene modification-driven development of capabilities to transport externally available metabolites into the cells and utilize these (Cui et al., 2023). It could also allow the loss of certain *de novo* biosynthesis capabilities through genomic mutations and deletions, as the resultant strain would be supported with supplements from the community (Li et al., 2016; Kelly et al., 2016a,b,c). The need to protect the cells from toxic products released from plant material biodegradation and to leverage physical association with a donor for better efficiency of nutrient acquisition is also likely a promoter of genomic changes. Genome evolution in the face of temporal changes in nutrient availability could make an organism either a specialist, thriving at a specific time or under specific physiochemical conditions, or a generalist.

The most striking case is the loss of components of the methanogenesis system, causing both simple and complex impacts on the energy metabolism of the organisms. The genomes of *Mbb. ruminantium*, *Methanomassiliicoccales* isolates ISO4-G1 and ISO4-H5, and *Thermoplasmatales* archaeon BRNA1 lack coenzyme M biosynthetic genes (*comADE*) (Figure 4), causing a need for exogenous supply of CoM for the growth of these organisms (Li, 2016); no such information is available for the *Methanomassiliicoccales* isolates ISO4-G11, RumEn M1, and RumEn M2 (Jeyanathan, 2010; Söllinger et al., 2016). Almost all methanogens carry CoM transporter genes, *ssuABC*, as reflected in their sensitivities to bromoethane sulfonate (BES), an analog of CoM (Santoro and Konisky, 1987; Zhang et al., 2000). The requirement for CoM for rumen methanogens has been known for a long time (Balch et al., 1979; Balch and Wolfe, 1979a,b; Lovley et al., 1984), and a CoM auxotroph has been used in a bioassay for this coenzyme (Balch et al., 1979; Balch and Wolfe, 1979a,b).

*Methanomassiliicoccales* ISO4-G1 genome lacks the uroporphyrinogen-III C-methyltransferase (*corA*) gene that is involved in F_430_ biosynthesis and the organism likely requires F_430_ for growth (Li, 2016; Figure 4). *Mbb. millerae* SM9 and *Mbb. olleyae* YLM1 genomes do not carry any of the biotin biosynthesis genes (Figure 4). However, both genomes encode a biotin transporter, BioY (Kelly et al., 2016a,b,c), suggesting an ability of biotin uptake from the environment; rumen fluid contains biotin (Midla et al., 1998; Fitzgerald et al., 2000; Zimmerly and Weiss, 2001; Bergsten et al., 2003). In pure cultures, methanogens harboring CoM biosynthetic genes grow faster than the respective CoM auxotrophic strains (Lovley et al., 1984). On the other hand, auxotrophy could give a competitive advantage to methanogen in the rumen, as it would not have to invest energy for biosynthesis activities.

### 4.3. Facilitation through horizontal gene transfer (HGT)

The instances of horizontal gene transfer (HGT) from bacteria to methanogens have been reported in numerous studies (Deppenmeier et al., 2002; Fournier and Gogarten, 2008; Lurie-Weinberger et al., 2012; Garushyants et al., 2015) though the transfer of methanogenesis genes to non-methanogenic species has not yet been reported (Gribaldo and Brochier-Armanet, 2006). A highly visible case of the former is the transfer of acetate kinase (*ackA*) and phosphotransacetylase (*pta*) genes from clostridia that provided acetoclastic methanogenesis capability in *Methanosarcina* (Fournier and Gogarten, 2008). In *Methanobrevibacter smithii*, a human gut-abundant methanogen species, over 15% of the genomic coding regions have bacterial characteristics (Lurie-Weinberger et al., 2012). For rumen methanogens, most of the transferred genes likely originated from organisms belonging to the Firmicutes phylum (Leahy et al., 2010; Kelly et al., 2016c). We describe below two examples of HGT events that likely helped methanogens to adapt to the rumen ecosystem.

#### 4.3.1. Association with a nutrient donor

As many as 294 genes of *Mbb. ruminantium* M1 have been postulated to be HGT-derived (Leahy et al., 2010), and most of these are for glycosyl transferases and adhesin-like proteins, which likely support *Mbb. ruminantium* to adapt in this environment (Samuel et al., 2007; Lurie-Weinberger et al., 2012; Shterzer and Mizrahi, 2015). In terms of the number of adhesin-like proteins encoded by the genome, this organism ranks first among the rumen methanogens, followed by *Mbb. millerae* SM9 (Figure 4). These values are consistent with the observed overall fitness in the rumen environment and the roles of adhesins in facilitating interaction with other ruminal guilds (Leahy et al., 2010; Ng et al., 2016; Wei et al., 2017).

In a co-culture experiment where *Mbb. ruminantium* was found to form aggregates with *Butyrivibrio proteoclasticus*, a Gram-positive rumen bacterium that degrades plant polysaccharides and forms butyrate, acetate, and hydrogen (Kelly et al., 2010), the levels of six adhesin-like proteins were enhanced in the methanogen (Leahy et al., 2010). A similar interaction of *Mbb. ruminantium* with rumen protozoa *Epidinium* and *Entodinium* (Ng et al., 2016) and rumen anaerobic fungi of the *Piromyces* genus has been documented, and in both cases, cell-to-cell attachments were clearly visualized (Wei et al., 2017). For the interaction with the protozoa, *Mbb. ruminantium* employs Mru_1499, an adhesin (Ng et al., 2016), and its association with *Piromyces* facilitates a high degree of biomass degradation, and methane and acetate formation (Wei et al., 2017). These findings call for further studies on the functional roles as well as the bacterial or protozoan targets for a large number of genes for adhesin-like proteins that have been bioinformatically identified in rumen methanogen genomes (Figure 4).

#### 4.3.2. Acquisition of tannin tolerance

Tannins, which are water-soluble polyphenols and originate from plants, denature and precipitate proteins, thereby preventing their degradation by microbes in the rumen (Westendarp, 2006). This action facilitates the passage of proteins to the small intestine, wherein the free proteins, detached from the tannin, are hydrolyzed to generate amino acids for use by the host animal. Tannins are not significantly toxic to ruminants but possess antimicrobial properties, and accordingly, have been used to treat diarrhea and control parasite infection (Westendarp, 2006; Cardoso-Gutierrez et al., 2021).

An observed post-feeding decrease in the methanogen population in the rumen has been thought to be due to the tannins (Fagundes et al., 2020), and direct inhibition of methanogens by these compounds have also been reported (Tavendale et al., 2005; Goel and Makkar, 2011). Yet, some of the rumen methanogens tolerate tannins, and this is likely due to HGT-derived genes for tannin-modifying enzymes (Kelly et al., 2016c). An example of such an enzyme is the tannin acyl hydrolase of *Mbb. millerae* SM9 which hydrolyzes the galloyl ester bond in tannins releasing gallic acid and glucose (Banerjee et al., 2012). This hydrolase occurs mostly in bacteria and fungi (Banerjee et al., 2012) and represents the first known tannase in a methanogen (Kelly et al., 2016c). It is highly homologous to the *Lactobacillus plantarum* enzyme (Kelly et al., 2016c).

## 5. Ecophysiology of rumen methanogens: lessons learned from community-based analyses

The early studies on the methanogens’ contributions to the conversion of feed into nutrients in ruminants were based on isolation, cultivation, and functional characterizations of rumen isolates. These efforts revolutionized the field of anaerobic microbiology and provided a first look into the rumen microbiome metabolism and respective roles in host physiology (Bryant and Burkey, 1953; Hungate, 1969; Henderson et al., 2015; Seshadri et al., 2018; Zehavi et al., 2018). However, the challenges of culturing strict anaerobes and the multiple auxotrophies of many of the rumen microbes and their metabolic dependence on community members hindered progress in the culture-dependent approach (Bryant and Burkey, 1953; Hungate, 1969; Henderson et al., 2015; Seshadri et al., 2018; Zehavi et al., 2018). Then, omics technologies brought a culture-independent approach toward an advanced assessment of the composition, metabolic potentials, and more importantly, *in situ* contributions of rumen methanogens (Tajima et al., 2001a,b; Luton et al., 2002; Tatsuoka et al., 2004; Friedrich, 2005; Janssen and Kirs, 2008; King et al., 2011; Henderson et al., 2015). We summarize below the progress and the gaps in these efforts.

### 5.1. Insights into methane emission phenotypes inferred from 16S rRNA-based community analyses

The development of small subunit rRNAs, 16S and 18S, as universal genomic markers for taxonomic identification of prokaryotes and eukaryotes, respectively, has revolutionized the field of microbial ecology (Pace, 1997). The community structure and relative abundance of each taxon in a rumen sample could be analyzed by amplifying and sequencing the hypervariable regions of 16S or 18S rRNA genes and comparing the sequence information with a reference database (Janssen and Kirs, 2008; Henderson et al., 2015). Then, the resultant community structure information could be associated with the observed events and phenotypes such as methane emission, VFA profile, and high- versus low-efficiency animals (Danielsson et al., 2017). Such analyses could help to identify and target the methanogens that contribute to high methane emissions for developing highly specific anti-methanogen interventions while limiting the effects on ruminant’s feed utilization efficiency and health.

Cattle with higher feed efficiencies, as measured in terms of the amount of milk produced or weight gain per kilogram of dry matter intake (DMI), emit about 30% less methane than others (Hernandez-Sanabria et al., 2012). A strong relationship also exists between methane production and residual feed index (RFI) (Herd and Arthur, 2009; Muro-Reyes et al., 2011). An RFI value, which is independent of animal production parameters, is calculated from the difference between an animal’s actual and predicted feed intake values where the prediction is based on the animal’s body weight and growth rate over a specified period (Nkrumah et al., 2006). Cattle with low and high RFI values are categorized as “efficient” and “inefficient,” respectively. The efficient animals eat less than the predicted average and produce less methane (Hegarty et al., 2007; Waghorn and Hegarty, 2011). Since methane emissions cause energy loss from the feed, high and low-methane-emitting animals are also classified as inefficient and efficient, respectively.

Supplementary Table S1 presents the observed relationships between methanogen abundance and methane emission phenotypes. In general, *Methanobrevibacter* spp. and *Methanosphaera* spp. were detected in higher abundance, numerically, in the rumen of high and low methane-emitting cattle, respectively (Kittelmann et al., 2014; Shi et al., 2014; Stepanchenko et al., 2023). High abundances of *Mbb. ruminantium* and unclassified *Methanomassiliicoccales* have been correlated to low emitting phenotype while that of *Methanobrevibacter gottschalkii* was associated with high methane phenotype (Danielsson et al., 2017). In contrast to the above findings, Wallace et al. (2015) reported that both *Methanobrevibacter* spp. and *Methanosphaera* spp. were enriched in the high methane emitter.

While CO_2_-hydrogenotrophs were found in both high and low methane emitters, total methanogen abundance was double in high methane emitters than in the low methane emitters (Auffret et al., 2018). An instance with a 7 times higher abundance of *Candidatus* Methanomethylophilus, a methyl-dismutating methanogen, in low-emitting animals than in high-emitting animals, has been reported (Auffret et al., 2018; Supplementary Table S1). In another case, the rumen microbiomes of both high and low methane emitters were found to exhibit similar abundances of methanogens, with *Mbb. gottschalkii* and *Mbb. ruminantium* as dominant members (Kittelmann et al., 2014); *Methanosphaera* spp. and members of *Methanomassiliicoccales* order were present at lower abundances. A higher value for the abundance of *Methanomassiliicoccaceae* has been recorded for the rumen of barley-fed beef steers with low RFI than with high RFI (Li and Guan, 2017). This mixed picture originates from the complexity of the rumen microbiome, variable feed composition, animal production systems, and sampling times, as well as the uncertainties in the 16S rRNA-based genotype assessments as detailed below. Of the available ruminant datasets, those pertaining to agriculturally important ruminants other than cattle (e.g., buffalo, yak, goat) and ruminants from the low- and middle-income countries are still limited, and this area needs more attention for further studies (Xie et al., 2021; Arndt et al., 2022).

Although the 16S rRNA amplicon sequence-based method is widely used in microbial community analysis and offers several advantages, it is important to consider the following limitations. First, the choice of a particular hypervariable region of 16S rRNA as the target of amplification influences the results’ accuracy. The often-used hypervariable region 4 (16S rRNA-V4) underrepresents methanogen species in the amplicons due to poor sequence homology (Supplementary Figure S1; Gilbert et al., 2014), and the V6-V8 regions, as well as archaeal-specific or degenerate primers (A109F/958R or 1Af/1100Ar), have been suggested as more effective tools for capturing rumen archaeome diversity (Janssen and Kirs, 2008; Tymensen and McAllister, 2012; Snelling et al., 2014; Li et al., 2016; Bahram et al., 2018). Accordingly, to analyze both ruminal bacterial and archaeal communities, the 16S rRNA primer set combinations targeting bacterial V1-V3 or V4 and archaeal V6-V8 regions have been used (De Mulder et al., 2016; Lopes et al., 2021; Tan et al., 2021).

Second is the accuracy of the reference taxonomy that determines the quality of classification (Schloss and Westcott, 2011), as a noticeable fraction of the sequences in the commonly used databases, RDP (Cole et al., 2014), SILVA (Quast et al., 2013) and Greengenes (DeSantis et al., 2006), lack informative annotation beyond the genus level. Consequently, the highest taxonomic confidence for the amplicon-based approach reaches only the genus level (Schloss and Westcott, 2011; Johnson et al., 2019). The outcomes can be improved by using curated niche-specific databases (Henderson et al., 2019). Such databases are available for rumen and bovine GIT (Kittelmann et al., 2014; Seedorf et al., 2014; Shi et al., 2014; Ritari et al., 2015), insect gut (Newton and Roeselers, 2012; Mikaelyan et al., 2015), freshwater (Rohwer et al., 2018) and marine ecosystems (Tangherlini et al., 2018), and wastewater treatment units (McIlroy et al., 2017). Third, DNA-based analyses cannot distinguish between active community members and non-active or even non-viable members. Lastly, a marker gene-based analysis does not provide information on the full genomes, and consequently, fails to reveal information on the metabolic capabilities of individual organisms, especially those lost through mutations or gained horizontally.

Even then, the 16S rRNA-based approach serves as an affordable and powerful tool for the initial analysis, providing encouragement for higher-resolution omics analyses toward a holistic picture of rumen microbiome processes that contribute to methane emissions from ruminants. A hopeful development is that the full-length rRNA gene sequences recovered from ecological samples are increasing the resolution for phylogenetic profiling (Matsuo et al., 2021). With latest advancements in the next-generation DNA sequencing technology, which substantially lowers the sequencing costs, shallow shotgun metagenomic sequencing could provide an alternative and effective method for characterizing microbiome samples. It offers both taxonomic and functional information at a cost comparable to amplicon-based 16S rRNA analysis (Hillmann et al., 2018; Xu et al., 2021; Stothart et al., 2022; La Reau et al., 2023).

### 5.2. Metabolic inferences from omics analyses

Shotgun metagenome and metatranscriptome sequencing, and metaproteomic and metabolomic analyses, stable-isotope probing, as well as full genomes of the isolates, have made it possible to perform thorough and precise *in situ* assessments of the structures and metabolic functions of the rumen microbiome (Shi et al., 2014; Estes et al., 2018; Stewart et al., 2018; Shakya et al., 2019; Wilkinson et al., 2020; van Cleef et al., 2021, 2022). The recently developed technology to rapidly generate full genome sequences from metagenomic DNA samples, namely metagenome-assembled genomes or MAGs (Tyson et al., 2004; Almeida et al., 2019; Nayfach et al., 2019; Youngblut et al., 2020; Haryono et al., 2022) has been extended to studies on rumen microbiome (Solden et al., 2018; Stewart et al., 2018, 2019; Wilkinson et al., 2020; Xie et al., 2021) and it allows the assignment of potential metabolic capabilities and *in situ* roles to microbes that have not even been obtained in pure or enrichment cultures.

Thousands of microbial MAGs have been recovered from the rumen samples (Solden et al., 2018; Stewart et al., 2018, 2019; Wilkinson et al., 2020; Xie et al., 2021). Two studies delivered >10,000 MAGs even from short-read sequences (Wilkinson et al., 2020; Xie et al., 2021). The genome sequences are facilitating not only the predictions of systems’ metabolic capabilities but also the strategy for genetic manipulations *in situ* (Roehe et al., 2016). Additionally, pangenome analysis from the MAG datasets is helping to identify environment-signature genes that could shed more insight into specific organism’s lifestyles and roles in an ecosystem, such as the rumen (Hansen et al., 2011; de la Cuesta-Zuluaga et al., 2021).

A major caveat of metagenomic analysis is its inability to distinguish between dead, dormant, and living cells (Shakya et al., 2019; Weinroth et al., 2022). It also fails to offer a complete assessment of the true *in situ* metabolic activities of the consortia (Shakya et al., 2019). It is only a combination of the genome and MAG sequences and metatranscriptomic, metaproteomic, and metabolomic data helps to assign comprehensive potential metabolic capabilities and capture real-time community metabolic activities and responses toward environmental changes such as feeding for the animals, and following are some of the examples of such studies (Shi et al., 2014; Li and Guan, 2017; Ma et al., 2018; Sollinger et al., 2018; Stewart et al., 2019; Wilkinson et al., 2020; Pitta et al., 2021; Xie et al., 2021; Pitta et al., 2022).

There are reports showing disagreements between the findings about rumen methanogens’ metabolic activities from DNA- and RNA-based characterizations (Shi et al., 2014; Li et al., 2016; Pitta et al., 2022) and the ratio of the number of transcripts and copies of the corresponding DNA (mRNA:DNA) has been proposed as an indicator of *in situ* metabolic activity (Pitta et al., 2022). Methanogens of the *Methanobacteriale*s order account for more than 61% of methanogen DNA sequences followed by *Methanomassiliicoccales* which contributes 15.8% of the sequences (Janssen and Kirs, 2008). In a study where the CO_2_-hydrogenotrophic *Methanobacteriale*s were highly represented in both the metagenomic and metatranscriptomic datasets, the respective mRNA:DNA value for formyl-MFR dehydrogenase, a CO_2_-reduction methanogenesis gene, was 1.5:1 (Pitta et al., 2022). In contrast, the mRNA:DNA value for *mtaB* of *Methanosphaera* species, which are methyl-hydrogenotrophs, was 6:1. A positive correlation between *Methanomassiliicoccales rRNA* and *mt*MA transcripts with the CH_4_ emission rate over time following feeding has also been recorded (Sollinger et al., 2018). These observations suggest that *Methanomassiliicoccales* and *Methanosphaera* species are more active than previously thought (Shi et al., 2014; Li et al., 2016; Sollinger et al., 2018; Söllinger and Urich, 2019; Pitta et al., 2021, 2022).

### 5.3. Hydrogen removal: inter- and intra-guild competitions

There have been efforts to determine the metabolic responses of specific methanogens to temporal $p_{H_{2}}$ changes in the rumen and link these to the organisms’ $p_{H_{2}-}$ thresholds or hydrogen affinities and deployment of specific enzymes (Sollinger et al., 2018; Feldewert et al., 2020; Pitta et al., 2022). Such details are needed for judicial targeting of methanogens for mitigating enteric methane emission. In this effort, the methyl-CoM reductase isozymes (McrI and McrII), and methanol and methylamine-specific methyl transferases have particularly been in focus.

**Figure 6:**
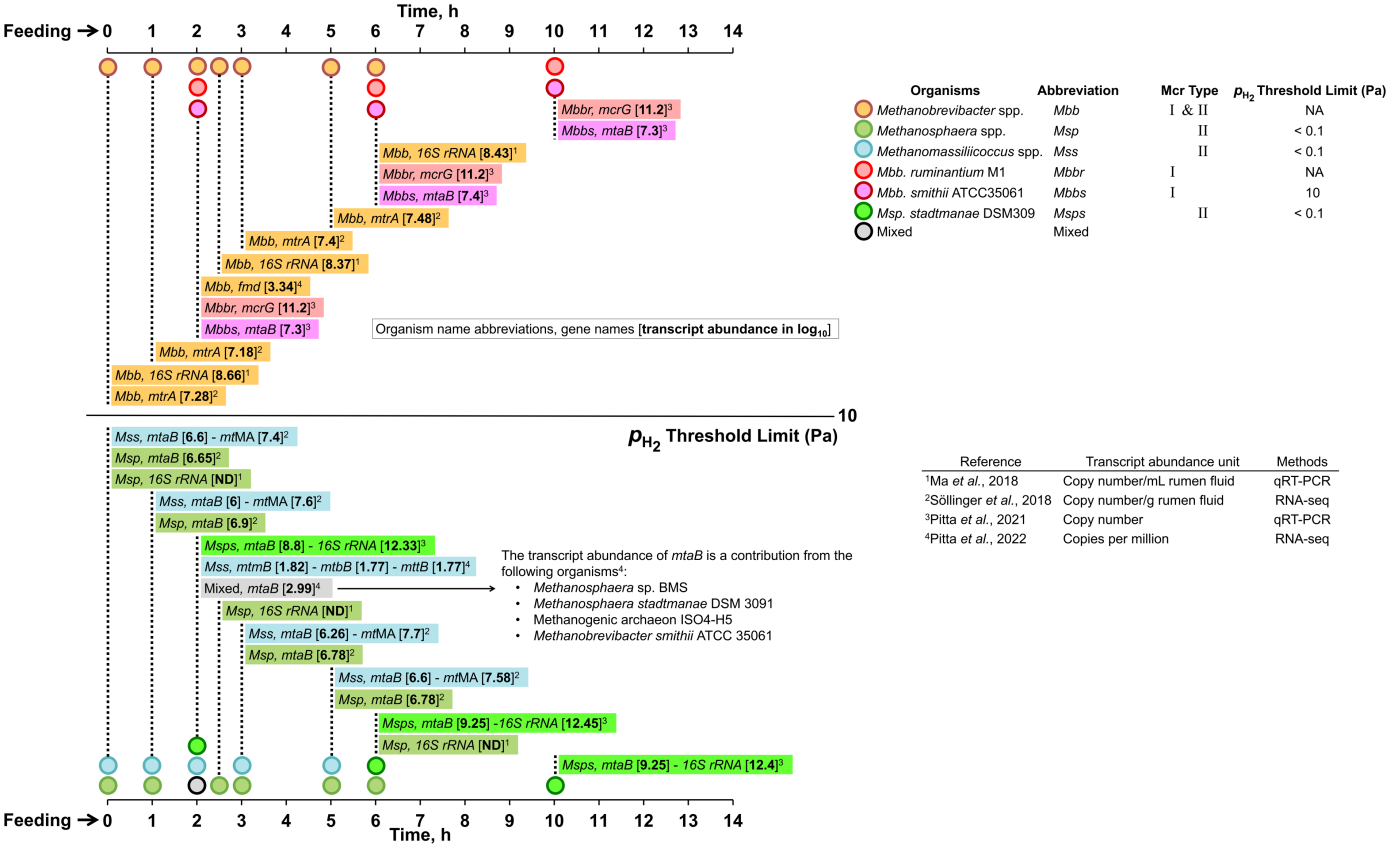
Temporal dynamics of rumen methanogen following feeding, a takeaway from transcriptomic studies. Transcript abundance of hydrogenotrophic and methyl-dismutating methanogenesis-related genes of rumen methanogens post-feeding. The abundance is shown in log_10_ values based on (Ma et al., 2018) or estimated from Sollinger et al. (2018) and Pitta et al. (2021, 2022), respectively. The $p_{H_{2}}$ threshold limit for *Mbb. smithii* (Feldewert et al., 2020). *fmd*, formylmethanofuran dehydrogenase; *mcrG*, methyl-CoM reductase subunit G; *mtaB*, methylcobamide:CoM methyltransferase; *mtmB*, monomethylamine methyltransferase; *mtbB*, dimethylamine methyltransferase; *mttB*, trimethylamine methyltransferase; *mt*MA, summarizes mono-, di-, and trimethylamine-specific methyltransferase (*mtmB*, *mtbB*, and *mttB*) transcripts whereas *mttB* transcripts constitute >70% of the *mt*MA transcripts; ND, not determined.

In *Methanothermobacter* species, McrI and McrII are encoded by the *mcrBDCGA* and *mrtBDGA* genes and expressed under low and high hydrogen availabilities, respectively (Rospert et al., 1990; Reeve, 1992; Morgan et al., 1997; Reeve et al., 1997), and these two systems are readily identified in methanogen genomes via protein primary sequence-based homology searches (Deppenmeier et al., 2002; Galagan et al., 2002; Fricke et al., 2006; Maeder et al., 2006; Jeyanathan, 2010; Leahy et al., 2010; Lambie et al., 2015; Li et al., 2016; Söllinger et al., 2016; Kelly et al., 2016a,b,c). As presented below and summarized in Figure 6, the reported data that links these factors together presents a complex and at times apparently contradicting picture.

Following feeding, the concentrations of fermentation products such as CO_2_, H_2_, and VFAs increase, and methyl-containing compounds (i.e., methanol, mono-, di-, and trimethylamines) become available in the rumen due to the resident microbiome’s metabolic activities (Sollinger et al., 2018; Kelly et al., 2019). This situation sets up a competition among various functional guilds of ruminal methanogens (Rooke et al., 2014; Sollinger et al., 2018; Martínez-Álvaro et al., 2020; Ungerfeld, 2020). For example, in one case, it was found that the abundances of *Methanosphaera* and *Methanomassiliicoccales* transcripts increased immediately after feeding (1–3 h), suggesting that the methanogenesis activity of methyl-hydrogenotrophs spikes following increased availability of hydrogen, methanol, and methylamines (Sollinger et al., 2018; Pitta et al., 2021). Methanogenesis activity of CO_2_-hydrogenotrophs such as *Methanobrevibacter* spp., however, remained constant (Sollinger et al., 2018; Pitta et al., 2021). In another case, significant activity of CO_2_-hydrogenotrophs has been observed at 6–10 h post-feeding (Pitta et al., 2021).

A theoretical analysis assuming all reactants, except H_2_, being under standard conditions, suggested that the thermodynamic equilibrium (Figure 5) of the CO_2_-hydrogenotrophic methanogenesis will be reached at a $p_{H_{2}}$ of 0.18 Pa, and for the methyl-hydrogenotrophic system, it would occur at a much lower $p_{H_{2}}$ of 8 × 10^−15^ Pa, suggesting competitive advantages of methyl-reducing members over CO_2_-reducing hydrogenotrophs at low $p_{H_{2}}$ . However, since in a typical rumen, $p_{H_{2}}$ is relatively high (162 Pa), the ecological success of the methyl-reducing hydrogenotrophs is more likely determined by their ability to utilize methyl-group containing substrates and the availability of these substrates in the rumen (Thauer et al., 2008; Feldewert et al., 2020).

Further adaptation has been observed following this common response toward $p_{H_{2}}$ , where methyl-hydrogenotrophs developed intra-guild competition and substrate preference. For example, a study documented that immediately after feeding, when rumen $p_{H_{2}}$ is high, the abundance of *Methanosphaera mtaB* mRNA abundance soared, while a similar response was seen for the *Methanomassiliicoccales mt*MA and not *mtaB* (Sollinger et al., 2018). This finding indicates that *Methanosphaera* and *Methanomassiliicoccales* were positioned to utilize two different methyl-group containing substrates, methanol, and methylamines, respectively, although both groups can utilize all these compounds.

## 6. Reflections and future outlooks

A detailed understanding of the diversity, lifestyle, and metabolism of rumen methanogens is key to developing strategies for achieving a substantial reduction of methane emissions from ruminants. The following section lays out key findings as well as challenges, research questions, and outlooks to guide future research toward the stated goal.

### 6.1. Evolutionary development of rumen methanogens and implications of their special properties for *in vitro* studies

Like other host-associated relatives, rumen methanogens have evolved from free-living ancestors through genome-size reduction, mutations, and gene acquisitions through horizontal gene transfer (HGT) (Shterzer and Mizrahi, 2015; Söllinger et al., 2016; Thomas et al., 2021). This genome streamlining process has provided competitive advantages to the rumen methanogens, allowing them to: (1) conserve energy through auxotrophies and transform into oligotrophic metabolic lifestyles and become metabolically efficient; (2) increase fitness through acquisitions of new metabolic capabilities; and (3) develop syntrophic interactions with hydrogen-producing bacteria and protozoa for effective transfer of H_2_. These very factors pose serious challenges to the isolation of methanogens for use in *in vitro* physiological studies (Seshadri et al., 2018; Zehavi et al., 2018). A report on *Methanomassiliicoccales* showcases potential of bias when employing artificial laboratory culturing conditions as these tend to enrich the metabolically versatile, free-living environmental members over the auxotrophic gut-associated species (Söllinger et al., 2016). For instance, the *Methanomassiliicoccus luminyensis* and *Methanomassiliicoccus intestinalis*, which were isolated from human feces, belong to an environmental clade and non-gut-associated cluster (Dridi et al., 2012; Borrel et al., 2013; Söllinger et al., 2016). Thus, future isolation efforts for rumen methanogens must leverage information on their metabolic dependencies and syntrophic lifestyles gathered from microbial community analyses (Seshadri et al., 2018; Zehavi et al., 2018).

### 6.2. Hydrogen removal and methane formation in the rumen – incomplete information on the molecular basis

There is a great need for basic information for deciphering the mechanisms driving hydrogen removal and methane formation in the rumen in the face of temporal fluctuations in $p_{H_{2}}$ and availability of methanol and methylamines and time of deployment of two types of hydrogenotrophs. The values for individual methanogen’s threshold of $p_{H_{2}}$ and affinity (K_s_ values) for CO_2_ and methyl-group containing substrates, respectively, and the efficiency of harvesting electrons from bacterial and eukaryotic syntrophic partners are in this list (Feldewert et al., 2020).

Methyl-reducing hydrogenotrophs belonging to the poorly characterized *Methanomassiliicoccales* order are of particular interest (Dridi et al., 2012; Gorlas et al., 2012; Borrel et al., 2013). These organisms have lost the genes for all enzymes catalyzing the first six steps of the CO_2_-reducing methanogenesis pathway, an unprecedented phenomenon that has not been encountered in any other methanogen order (Dridi et al., 2012; Li et al., 2016; Kelly et al., 2016a,b; Lyu and Liu, 2019). However, they carry genes for the utilization of a diversity of methylated compounds, suggesting their metabolic limitation on one side and versatility on the other side as a way of adaptation to a nutrient-rich environment (Söllinger et al., 2016; Thomas et al., 2021).

An anticipated greater contribution of the previously underestimated *Methanomassiliicoccales* to methane production in the rumen (Pitta et al., 2022) is potentially driven by two factors. First, their lower threshold for H_2_ as mentioned above allows them to function at lower $p_{H_{2}}$ than that of the CO_2_-reducing hydrogenotrophs. Second, by utilizing a diversity of methyl groups containing methanogenesis substrates effectively, they prevail over other methyl-reducing hydrogenotrophs and methyl-dismutating methanogens. Nevertheless, a more definitive assessment of such relative capabilities requires information on the affinities (K_s_) for methyl-group containing substrates of methanogens that utilize methyl groups for methanogenesis.

There is a lack of sufficient data for assigning the $p_{H_{2}}$ conditions under which an organism will deploy a particular Mcr isoenzyme. As a result, the reported assignments do not always match with an observed physiological response of the respective organisms toward hydrogen availability. For example, as mentioned above, *Methanomassiliicoccales* and *Methanosphaera* spp. employ Mcr II that is thought to be expressed under high $p_{H_{2}}$ conditions (Rospert et al., 1990; Reeve, 1992; Morgan et al., 1997; Reeve et al., 1997), and this functional association contradicts the observed lower $p_{H_{2}}$ threshold of these organisms (Feldewert et al., 2020). This discrepancy illuminates a major gap in studies on an enzyme that is the ultimate biological producer of methane (Wolfe, 1992).

The information on Mcr isoenzymes originated from investigations with two *Methanothermobacter* species, which are thermophiles (Rospert et al., 1990; Reeve, 1992; Morgan et al., 1997; Reeve et al., 1997), and these may not apply to other methanogens. The suggestion that even the activity of a Mcr could be under $p_{H_{2}-}$ or redox-based regulation (Susanti et al., 2014) has also not been tested. As a result, a primary sequence homology-based identification of *mcrA* and *mrt*, which is the norm in ecological work, cannot indicate with certainty if the enzymes encoded by these genes are expressed or active under a particular $p_{H_{2}}$ condition.

The possibility that certain *Methanobrevibacter* species (i.e., *Mbb. ruminantium* M1) may perform methyl-hydrogenotrophy employing an HGT-derived methyl transferase (Leahy et al., 2013) brings a new dimension to the roles of these organisms in the rumen. Also, right after feeding, the H_2_ production rate far exceeds rumen methanogens’ available capacities to utilize this energy source (Rooke et al., 2014; Ungerfeld, 2020). This situation sets a lag between H_2_ production and CH_4_ emission (Rooke et al., 2014; van Lingen et al., 2017), and the suggestion that this effect is mainly due to a delayed expression of methanogenesis genes needs a detailed interrogation (Sollinger et al., 2018; Ungerfeld, 2020).

### 6.3. Methanogenesis from formate in the rumen – largely untapped area of research

Early studies showed that formate is not a major precursor of methane in the rumen (Carroll and Hungate, 1955; Hungate et al., 1970) and this conclusion has recently been supported by the observation that the rumen samples lack transcripts for formate dehydrogenase; Fdh (Pitta et al., 2022) and formate were not detected at most timepoints following feeding (Sollinger et al., 2018). These findings contrast the observation that *Methanobrevibacter* species represent 60–80% of the rumen methanogen community (Henderson et al., 2015), and as mentioned above, these organisms carry the *fdhABC* genes (Schauer and Ferry, 1982; Nölling and Reeve, 1997). Fdh is encoded by an *fdhABC* operon that provides FdhAB and FdhC as a catalytic unit and formate transporter, respectively (Baron and Ferry, 1989). FdhAB oxidizes formate to CO_2_ and utilizes the electrons so generated for the reduction of F_420_ to F_420_H_2_.

Indeed, in *Mbb. ruminantium* M1, the abundance of *fdhAB* rRNA is enhanced when this methanogen is grown in a co-culture with *Butyrivibrio proteoclasticus* B316, an H_2_ and formate producer, indicating formate utilization by this methanogen during this syntrophic growth (Leahy et al., 2010). Also, formate as a methanogenesis substrate supports the growth of *Mbb. ruminantium* (Smith and Hungate, 1958). The sheep rumen microbiome has been found to exhibit poor expression of bacterial formate hydrogen lyases and other formate dehydrogenases (Greening et al., 2019). This suggests that formate produced in the rumen would be available for formate utilizers like *Methanobrevibacter*. The formate metabolism could also bring ecological fitness to the *Methanobrevibacter* spp. and *Mb. formicicum*.

The absence of formate dehydrogenase in the methyl-hydrogenotrophs gives free rein to formate utilizing methanogens for this substrate. In addition, being soluble, formate is an excellent vehicle for interspecies electron transfer and planktonic metabolism (Thiele and Zeikus, 1988; Leng, 2014), and removal of formate would prevent the acidification of the system as the pK_a_ of the formic acid/formate pair is 3.75. The reported low levels of formate and *fdh* transcripts in the rumen (Sollinger et al., 2018; Pitta et al., 2022) could be rationalized by the high abundance of the *Methanobrevibacter* population. Also, the reported data were collected 2 h after feeding (Pitta et al., 2022), where the formate level would have dropped substantially, obviating the need for high-level *fdh* transcripts. The identification of formate utilizing methanogens in early colonizers in calves (Guzman et al., 2015) is intriguing, raising a question of whether formate is the substrate for methanogenesis at this stage. Further study on the formate and dissolved H_2_ levels in the undeveloped foregut of pre-ruminants could give insights into the role of methanogenesis from formate at this stage of the animals.

Thus, formate methanogenesis is an important yet less appreciated area of rumen microbial metabolism research. It needs to be studied with consideration that acetogenic bacteria with their ability to perform acetogenesis with formate would compete for this substrate (Greening and Leedle, 1989; Doré and Bryant, 1990; Schink et al., 2017; Greening et al., 2019; Moon et al., 2021).

### 6.4. Harnessing omics approach for analyzing metabolism of rumen methanogens – current status and future steps

As mentioned above, there is a need to strengthen the 16S rRNA sequence database as it would allow effective use of the most affordable route to community composition analysis that employs sequencing and analysis of short (~100–200 bp) amplicons of 16S rRNA gene (Johnson et al., 2019; Weinroth et al., 2022). Under the current situation, the results of such analyses need to be considered with caution as it often provides only low-resolution identities, an over-simplification of the diversity and incomplete metabolic inferences for methanogens in the rumen (Pitta et al., 2022). There are instances where metatranscriptomic and 16S rRNA amplicon sequences from rumen samples detected the presence of *Methanocaldococcus* spp. and *Methanopyrus* spp., which are obligate hyperthermophiles (Lyu and Liu, 2019), and *Mbb. smithii*, a human-associated organism (Zhou et al., 2009; Kong et al., 2013; Auffret et al., 2018; Mann et al., 2018; Tan et al., 2021).

Comprehensive and effective comparative genomic studies and analysis of metatranscriptomic and metaproteomic data with rumen methanogens are limited by the inadequate number of well-annotated reference genomes of pure culture isolates and MAGs. Even the Hungate 1,000 Project which generated sequences of 501 genomes, covering 480 ruminal bacteria and 21 archaea species, represents only 15 rumen methanogens (Seshadri et al., 2018). The number of isolate genome and MAG sequences for rumen methanogens that are publicly available are only 14 (Supplementary Table S2) and 206, respectively (Söllinger et al., 2016; Stewart et al., 2018, 2019; Wilkinson et al., 2020; Glendinning et al., 2021; Xie et al., 2021).

The gap in reference data extends beyond the molecular data. In most cases there is little information on the association of isolate genomes, MAGs, and even sometimes the metatranscriptomic and metaproteomic data for the methanogens with the following key parameters: (i) details of the feed; (ii) spatial location within the rumen, namely, fiber-associated, planktonic, and epimural microbiome; (iii) co-occurrence, such as association with the syntrophic partners, protozoa, and bacteria; (iv) timing of sampling with respect to feeding; and (v) comparison with free-living counterparts. In a recent study with cattle grazing tall fescue, a major perturbation of the microbiome by a toxic version of the grass was detected only when the sessile and planktonic fractions were analyzed separately (Khairunisa et al., 2022) and similar observations have been reported by others (Pitta et al., 2014).

The developmental stage of the animal host is a key factor (Fonty et al., 1987; Morvan et al., 1994; Fonty et al., 2007; Guzman et al., 2015; Friedman et al., 2017; Furman et al., 2020), as rumen microbiome modulation at this early stage of the animals is being considered as a potential methane mitigation strategy (Meale et al., 2021). The metabolism of methanogens that colonize the gut of the pre-ruminant phase and its influence on the development of the rumen remains to be investigated critically.

### 6.5. Future steps

This review shows that the current knowledge of rumen methanogens cannot adequately support the efforts for designing measures that will mitigate methane emissions from ruminants and preserve rumen function in the absence or in reduced methanogenic activity. Even after ~80 years of research, it is not known why *Methanobrevibacter* spp. dominate the rumen microbiome and what their specific contributions are. Filling these gaps requires significant isolation efforts, especially for those members with very few or no pure culture representatives (e.g., *Methanomassiliicoccales*) and the generation of more well-annotated genomes and MAG sequences.

A culturomic approach leveraging both undefined media containing rumen fluid and defined media showed that 23% of the rumen microbiota is cultivable with these technologies (Zehavi et al., 2018). However, it provided a relatively low coverage for the rumen methanogens. For example, of the prokaryotes in the Hungate 1,000 culture collection, methanogens represent only 4.1% of the total (Seshadri et al., 2018). Thus, for an isolation effort to be productive will require innovative approaches. If the unknown growth requirements make it difficult to generate axenic cultures, attempts could be made to obtain low-complexity mixed cultures. Since 16S rRNA provides an affordable and amenable route for rapid assessment of microbiome diversity, the respective database needs to be strengthened.

With more reference isolates, comprehensive physiological studies could occur with a focus on newly recognized genomic features that promote colonization of the rumen and high-level methane production. One high-value area is the cellular interactions of rumen methanogens with their syntrophic partners such as protozoa, fungi, and bacteria where the following questions are key. *What governs such interactions? What defines the specificity and recognition by interacting partners? What are the mechanisms of interspecies electron transport?* Co-occurrence analysis that could reveal metabolic differences between host-associated and free-living methanogens would also be valuable. Detailed information on methanol and methylamine concentrations in the rumen of animals fed various diets, thresholds for these substrates and $p_{H_{2}}$ of various rumen methanogens, and catalytic properties and expression conditions of the Mcr isoenzymes are needed to make the analysis and interpretation of *in situ* observations more reliable. The information on the Mcr isoenzymes is also needed for correct functional annotations of *mcr* and *mrt* homologs. The diversity and metabolic activities of methanogens residing in various locations of the rumen as mentioned above could give insights into true activities driving *in situ* methane production.

## Author contributions

BK: Data curation, Formal analysis, Writing – original draft, Writing – review & editing. CH: Data curation, Formal analysis, Writing – original draft, Writing – review & editing. KI: Formal analysis, Writing – original draft. BM: Formal analysis, Funding acquisition, Supervision, Writing – review & editing. DS: Conceptualization, Formal analysis, Writing – original draft, Funding acquisition, Supervision, Writing – review & editing, Data curation.
