# Supplementary material for: Evolving understanding of rumen methanogen ecophysiology
Source: Front Microbiol. 2023 Nov 6;14:1296008. doi: 10.3389/fmicb.2023.1296008 (PMC10658910; doi:10.3389/fmicb.2023.1296008)
Supplement: Supplementary file 1 [file Data_Sheet_1.docx]

**SUPPLEMENTARY MATERIALS**

**Evolving Understanding of Rumen Methanogen Ecophysiology**

Bela Haifa Khairunisa^1,†^, Christian Heryakusuma^2,3,†^, Kelechi Ike^4^,

Biswarup Mukhopadhyay^2,3,5,*^, Dwi Susanti^1,*^

^1^Microbial Discovery Research, BiomEdit, Greenfield, IN 46140, USA

^2^Genetics, Bioinformatics, and Computational Biology Ph.D. Program, Virginia Tech, Blacksburg, VA 24061, USA

^3^Department of Biochemistry, Virginia Tech, Blacksburg, VA 24061, USA

^4^Department of Biology, North Carolina Agricultural and Technical State University, Greensboro, NC 27411, USA

^5^Virginia Tech Carilion School of Medicine, Virginia Tech, Blacksburg, VA 24061, USA

^†^These authors contributed equally to this work and share first authorship

***Correspondences:**

Dwi Susanti

[dsusanti@biomedit.com](mailto:dsusanti@biomedit.com)

Biswarup Mukhopadhyay

[biswarup@vt.edu](mailto:biswarup@vt.edu)

**Supplementary Methods**

**16S ribosomal RNA gene-based phylogenetic tree reconstruction**

16S ribosomal RNA gene sequences were obtained from the National Center for Biotechnology Information (NCBI) database and RIM-DB [1]. The phylogenetic tree was reconstructed by using a web based NGPhylogeny server (<https://ngphylogeny.fr/>) [2, 3]. In this web server, a semi-automatic pipeline was chosen and FastME/OneClick workflow was selected. In the FastME/OneClick workflows, Multiple Alignment using Fast Fourier Transform (MAFFT), Block Mapping and Gathering with Entropy (BMGE), FastME, and Newick display were used for multiple sequence alignment, alignment curation, tree reconstruction, and tree rendering, respectively. Once DNA sequences were uploaded as input data, the parameter for FastME was changed to include bootstrap branch supports with the number of replicates of 1000. MAFFT, BMGE, and Newick display were set to default parameters. The phylogenetic tree was visualized using FigTree v1.4.4 (<http://tree.bio.ed.ac.uk/software/figtree/>).

**Figure S1. A multiple sequence alignment of hypervariable regions of the 16S ribosomal RNA genes of select methanogenic archaea.** A 16S ribosomal RNA (rRNA) gene-based alignment was constructed following a protocol described in the Supplementary method. The nine hypervariable regions of V1 through V9 span nucleotides 69-99, 137-242, 433-497, 576-682, 822-879, 986-1043, 1117-1173, 1243-1294, and 1435-1465, respectively [4, 5]; numbering is based on the *E. coli* rRNA genes [4]. V9 region was not detected in all sequences. The names highlighted in yellow represent rumen-associated methanogens. The black bars under the multiple sequence alignment represent the consensus sequences. A higher black bar indicates a high degree of sequence conservation. Primers as shown with horizontal arrows and names target specific regions of 16S rRNA gene [6].

**Supplementary Tables**

**Table S1.** Methanogens associated with high and low methane-emitting animals.

| **Classifying parameter** | **Gene marker** | **Methane emission phenotype** | | **Study description**  **(Dairy/Beef, number of animals, feed)** | **Ref.** |
| --- | --- | --- | --- | --- | --- |
|  |  | **High (Inefficient)** | **Low (Efficient)** |  |  |
| CH_4_–IR | V4-16S rRNA^a^ | *Methanobrevibacter* SGMT clade, *Mbb. gottschalkii* | *Methanobrevibacter* RO clade, *Mbb. ruminantium;* and unclassified *Methanomassilicoccales* | Dairy, 73, silage (timothy, fescue, perennial ryegrass, red clover) and concentrate | [7] |
| CH_4_-RC | ssrRNA, 16S rRNA^b^ | *Mbb. gottschalkii* | *Methanosphaera* spp. | Sheep, 22, pelleted lucerne (alfalfa) diet | [8] |
| CH_4_-RC | 16S rRNA^b^ | Not reported | *Methanosphaera* spp. | Sheep, 118, pelleted lucerne | [9] |
| CH_4_-RC | 16S rRNA^c^ | *Methanobrevibacter spp. (94%), Methanobacterium, Methanococcus, Methanoculleus* | *Candidatus Methanomethylophilus* | Beef, 50, two diets: high and low forage | [10] |
| CH_4_-RC | 16S rRNA^d^ | *Methanobrevibacter spp, Methanosphaera spp* | Not reported | Beef, 72, two diets: high and medium concentrate | [11] |
| CH_4_-RC | 16S rRNA^e^ | *Methanobrevibacter spp* | *Methanosphaera spp* | Dairy, 10, silage- and concentrate-based TMR | [12] |
| **Classifying parameter** | **Gene marker** | **Residual Feed Intake (RFI** | | **Study description**  **(Dairy/Beef, number of animals, feed)** | **Ref.** |
|  |  | **High (Ineffficient)** | **Low (Efficient)** |  |  |
| RFI | 16S rRNA^f^ | *Msp. stadtmanae, Methanobrevibacter sp.* AbM4*, Methanobrevibacter sp.* 30Y-like*, Mbb. wolinii*-lik*e, Methanobacteriales*-like | *Mbb. smithii* SM9-like*, Mbb. smithii* PS-like*, and Methanobrevibacter sp.* FM1-like | Beef, 58, high concentrate oats-based diet | [13] |
| RFI | 16S rRNA^g^ | *Methanobrevibacter spp* | *Methanomicrococcus spp* | Beef, 27, high forage | [14] |
| RFI | 16S rRNA^h^ | *Methanobrevibacter ruminantium* | *Methanomassiliicoccales spp.* | Beef, 180, high energy diet (oats, barley, alfalfa pellets, and feedlot supplement) | [15] |

CH_4_-IR or CH_4_-RC, methane emission activity measured using an infrared-based analyzer or respiratory chamber, respectively; RFI, residual feed index [13, 14, 16, 17].

^a^Primer (name): forward (515′F), 5’-GTGBCAGCMGCCGCGGTAA-3’; reverse (805R), 5’-GGACTACHVGGGTWTCTAAT-3’ [13]

^b^Primer (name): forward (Ar915aF), 5′-CCATCTCATCCCTGCGTGTCTCCGACTCAGTCAGGAATTGGCGGGGGAGCAC-3′;

reverse (Ar1386R), 5′-CCTATCCCCTGTGTGCCTTGGCAGTCTCAGNNNNNNNNNNNNCAGCGGTGTGTGCAAGGAGC-3′ [18]

^c^Analysis was performed using shotgun metagenomic dataset and sequences were aligned to Greengenes and Kraken databases [10].

^d^Primer (name): forward (UniF), 5’-GTGSTGCAYGGYYGTCGTCA-3’; reverse (UniR), 5’-ACGTCRTCCMCNCCTTCCTC-3’ [19]

^e^Primer (name): forward (i958aF), 5′-AATTGGAKTCAACGCCKGR-3′; reverse (i378aR), 5′-TGTGTGCAAGGAGCAGGGAC-3′ [12]

^f^Primer (name): forward (Met 86f), 5’-GCTCAGTAACACGTGG-3’; reverse (Met 915r), 5’-GTGCTCCCCCGCCAATTCCT-3’ [20, 21]

^g^Primer (name): forward (Ar915aF), 5’-AGGAATTGGCGGGGGAGCAC-3’; reverse (Ar1386R), 5’-GCGGTGTGTGCAAGGAGC-3’ [13]

^h^16S rRNA sequences were assembled from RNA-Seq data and then mapped to a custom reference database for the V6-V8 regions for rumen-specific [archaea](https://www.sciencedirect.com/topics/immunology-and-microbiology/archaeon) [15]

**Table S2.** Isolates and genomes of rumen methanogen and close relatives.

| **Methanogen Species** | **Available isolate** | **Gold ID,**  **NCBI ID** | **Genome Completion Status** | **Genome Size (Mbp)** | **Mean relative abundance in ruminants [22]** | **Data type/**  **Origin** | **Ref(s)** |
| --- | --- | --- | --- | --- | --- | --- | --- |
| **Family: *Methanobacteriaceae*** |  |  |  |  |  |  |  |
| **Genus: *Methanobrevibacter (Mbb)*** |  |  |  |  |  | 16S rRNA and MAGs | [23] |
| *Mbb sp.* | Abm4*, | Gp0021414, CP004050.1 | F | 2 |  | Genome/Sheep abomasal isolate | [24] |
| *Mbb ruminantium* | M1 or DSM1093,  YE286 | Gp0002311, CP001719.1  Gp0035230, NA | F  P | 2.9,  1.8 | 27.1% | 16S rRNA, MAGs, transcriptome, and qRT PCR | [22, 25-28] |
| *Mbb boviskoreani* | JH1 | Gp0035818, BAGX00000000.2 | P | 2 | 0.9% | 16S rRNA, and MAGs | [22, 25, 26, 29] |
| *Mbb millerae* | SM9,  DSM 16643 or ZA-10,  HW02 | Gp0007703, CP011266.1 Gp0087971, SAMN02910315  Gp0096056, PRJNA252037 (no available sequence) | F  P  I | 2.5,  2.7,  - |  | 16S rRNA | [30],  [25, 31],  - |
| *Mbb olleyae* | YLM1,  DSM 16632 or KM1H5-1P | Gp0007263, CP014265.1  Gp0087972, SAMN02910297 | F  P | 2.2,  2.12 |  | 16S rRNA | [32],  [25, 31] |
| *Mbb gottschalkii* | HO or DSM11977*,  PG or DSM11978* | Gp0290545, SAMN10363317  Gp0127403, SAMN05216439 | P  P | 1.87,  1.86 | 46.7% | 16S rRNA, transcriptome | [22, 25, 26] |
| *Mbb thaueri* | CW or DSM11995* | Gp0113775, NA | P | 2.2 |  | 16S rRNA | [25, 33] |
| *Mbb wolinii* | SH* | Gp0047017, SAMN02744021 | P | 2 | 1.0% | 16S rRNA | [22, 25] |
| *Mbb acididurans* | DSM 15163* | Gp0115253, NA | P | 6.1 | 0% | 16S rRNA | [22] |
| *Mbb smithii* | DSM 2375*,  F1 or DSM 2374* | Gp0003638, SAMN00008835  Gp0003674, SAMN00008834 | P  P | 1.7,  1.7 | 0.5% | 16S rRNA and qRT PCR | [22, 25] |
| *Mbb woesei* | GS or DSM11979* | Gp0113776, NA | P | 1.5 |  | 16S rRNA and transcriptome | [25] |
| *Mbb sp.* | YE315 | Gp0118019, CP010834 | P | 2.27 |  | 16S rRNA | [25, 34] |
|  |  |  |  |  |  |  |  |
| **Genus:**  ***Methanosphaera (Msp)*** |  |  |  |  |  |  |  |
| *Msp sp.* | Group 5**,  A4,  ISO3-F5,  RUG761 [23]**,  WGK6**,  BMS** | -,  -,  -,  Gp0304284, ONYO00000000.1  Gp0107741, SAMN03108761  Gp0119560, CP014213 | P, M  P  P | -,  -,  -,  1.6,  1.7,  2.8 | 2.1%,  0.1%,  5.7%,  -,  -,  - | MAGs, 16S rRNA | [22, 23, 26, 35] |
| *Msp stadtmanae* | DSM 3091* | Gp0000406, CP000102.1 | F | 1.7 |  | 16S rRNA and qRT PCR |  |
| *Msp cuniculi* | 1R-7* | Gp0322644, SAMN04229037 | P | 1.9 | 0.2% | 16S rRNA; Rabbit intestinal tract isolate | [22, 36] |
|  |  |  |  |  |  |  |  |
| **Genus:**  ***Methanobacterium (Mb)*** |  |  |  |  | 0.6% |  | [22] |
| *Mb formicicum* | BRM9 | Gp0007264, CP006933.1 | F | 2.4 | 0% | 16S rRNA | [22, 37, 38] |
| *Mb bryantii* | M.o.H or DSM 863,  YE299 | Gp0322642, SAMN04229035 | P | 3.45 |  |  | [36] |
|  |  |  |  |  |  |  |  |
|  |  |  |  |  |  |  |  |
| **Family: *Methanosarcinaceae*** |  |  |  |  |  |  |  |
| **Genus: *Methanosarcina*** |  |  |  |  |  |  |  |
| *Methanosarcina mazei* | Go1 or DSM 3647* | Gp0000684, AE008384.1 | F | 4 | 0% | 16S rRNA | [22] |
| *Methanosarcina sp.* | Ms 97 or DSM 11855* | Gp0087973, SAMN02910340 | P | 3.1 |  |  | [39] |
| *Methanosarcina horonobensis* | HB-1*,  JCM 15518* | Gp0074649, CP009516.1  Gp0093132, SAMD00000526 | F  P | 5,  4.9 | 0% | 16S rRNA | [22] |
| *Methanosarcina barkeri* | CM1 | Gp0007672, CP008746 | F | 4.5 |  |  | [25, 38, 40] |
|  |  |  |  |  |  |  |  |
| **Genus: *Methanimicrococcus*** |  |  |  |  |  |  |  |
| *Methanimicrococcus blatticola* | PA*,  DSM 13328* | Gp0156044, NA  Gp0251965, SAMN08769569 | P  P | 1.77,  1.78 | 0.6% | 16S rRNA | [22, 26] |
|  |  |  |  |  |  |  |  |
|  |  |  |  |  |  |  |  |
| **Family: *Methanomicrobiaceae*** |  |  |  |  |  |  |  |
| **Genus: *Methanoculleus*** |  |  |  |  |  |  |  |
| *Methanoculleus sp.* | - | - | - | - | 0% | 16S rRNA | [22] |
| *Methanoculleus bourgensis or olentangyi [41]* | KOR-2 | - | - | - |  |  | [42] |
|  |  |  |  |  |  |  |  |
| **Genus:**  ***Methanomicrobium*** |  |  |  |  |  |  |  |
| *Methanomicrobium mobile* | BP, DSM1539 | Gp0047018, SAMN02745537 | P | 1.7 | 0.7% | 16S rRNA | [22, 25, 38, 43] |
|  |  |  |  |  |  |  |  |
|  |  |  |  |  |  |  |  |
| **Family: *Methanomassiliicoccaceae*** |  |  |  |  |  |  |  |
| Candidatus *Methanomethylophilus alvus* |  | Gc0042696 | F | 1.66 | 0.8% | 16S rRNA | [22] |
| Candidatus *Methanomethylophilus* | RUG779,  hRUG898 | Gp0304284, SAMEA104154002  Gp0326514, SAMEA104567052 | M, P  M, P | 1.26,  1.38 |  |  | [23] |
| *Methanomassiliicoccaceae* Group 4 | MpT1 | - | - | - | 1.0% | 16S rRNA | [22] |
| *Methanomassiliicoccaceae* Group 10 |  | - | - | - | 3.0% | 16S rRNA and MAGs | [22, 26] |
| *Methanomassiliicoccaceae* Group 11 | ISO4-G1 | Gp0139499, CP013703.1 | P | 1.59 | 0.2% | 16S rRNA and MAGs | [22, 25, 26, 35, 44] |
| *Methanomassiliicoccaceae* Group 11 | CRM1 | - | - | - | 0.2% | 16S rRNA | [22] |
| *Methanomassiliicoccaceae* Group 12 | ISO4-H5 | Gp0125684, CP014214 | F | 1.9 | 6.5% | 16S rRNA and MAGs | [22, 25, 26, 45] |
| *Thermoplasmatales archaeon* | BRNA1 | Gp0045376, CP002916.1 | F | 1.46 | 0% | 16S rRNA and MAGs | [22, 25, 26, 35] |
| *Methanomassiliicoccales* archaeon | RumEn M1*** | Gp0139355,  GCA_001421185.1 | D | 2.12 | 0% | Metagenome | [46] |
| *Methanomassiliicoccales* archaeon | RumEn M2*** | Gp0139355,  GCA_001421175.1 | D | 1.28 | 0% | Metagenome | [46] |
|  |  |  |  |  |  |  |  |
|  |  |  |  |  |  |  |  |
| **Family: *Methanotrichaceae*** |  |  |  |  |  |  |  |
| **Genus: *Methanothrix*** |  |  |  |  |  |  |  |
| *Methanothrix (Methanosaeta) concilii* |  | - | - | - | 0% | 16S rRNA | [22] |
|  |  |  |  |  |  |  |  |

*, non-rumen; **, MAG; ***, metagenome. Each of the Gold ID showed an available genome sequence of a rumen methanogen strain with the following genome completion status: P, permanent draft; F, finished; D, Draft; I: incomplete; MAG: metagenome-assembled genome. Rumen isolate information can be found in Table 1.

**Table S3.** Rumen methanogen abundance as affected by various diets.

| Diet composition item (% value) | Cattle  type | Methods and markers used | Methanogen abundance (%) | | | | | | | | | | | | | | Ref |
| --- | --- | --- | --- | --- | --- | --- | --- | --- | --- | --- | --- | --- | --- | --- | --- | --- | --- |
|  |  |  | *Methanobrevibacter* | | | | | | | | | *Methanosphaera* | | *Mcm* | *Thr* | Un |  |
|  |  |  | SGMT clade | | | | RO clade | | Other species | | |  |  |  |  |  |  |
|  |  |  | *M. smi* | *M. got* | *M. mil* | *M. tha* | *M. rum* | *M. oll* | *M. wol* | *M. woe* | *M. sp* | *M. std* | *Ms. sp* |  |  |  |  |
| Corn silage (24.5), alfalfa hay (16.7), corn meal (16.6), soybean meal (10.7), beet pulp (3.5), DDGS (3.5), molasses (3), mineral/vitamins (6.3) | Dairy | Sequencing V3-V4 region of archaeal 16S rRNA^a^ and qPCR analysis targeting a ~800 bp segment of *mcr*A^b^ |  |  |  |  |  |  |  |  | 86.9 |  | 0.8 | 10.4 | 0.4 |  | [47] |
| Corn silage (51.2), haylage (8.3), hay (13.4), concentrate (27.2) | Dairy | Sequencing of V1-V3 region of archaeal 16S rRNA^c,d^  and qPCR targeting a ~350 bp segment of *mcr*A^e^ | 28.55 | 0.04 | 10.99 | 30.70 | 26 |  |  | 2.12 |  |  | 0.41 |  |  |  | [48] |
| Corn silage (35), second-cut haylage (33), hay (72), canola meal (13.2), and soybean meal (19.8) | Dairy | Sequencing of a ~1260 bp of methanogen specific 16S rRNA^f^ |  |  |  |  | 96 |  | 1 |  |  | 2 |  |  |  |  | [49] |
| Wheat straw-based diet comprised of roughage (60), concentrate (40) | Beef | Sequencing of a ~1260 bp segment of 16S rRNA^f^ and a ~470 bp segment of *mcr*A^g^ |  |  |  |  |  |  |  |  | >83 |  |  |  |  |  | [50] |
| Perennial Ryegrass, white clover | Beef | Sequencing of the V6-V8 (∼492 bp) segment 16S rRNA^h^ |  | 43 |  |  | 33 |  |  |  |  |  | 14 |  |  |  | [51] |
| Spring barley (21.8), rape seed cake (22.2), clover grass silage (30.4), corn silage (24.5) | Dairy | Sequencing random 200 and 220 bp subsegments of 16S rRNA^i^ |  |  |  |  |  |  |  |  | 37.85 |  | 2.84 |  | 52.3 | 7 | [52] |
| Rhodes grass/*Chloris gayana* (15) and a sorghum high-grain mixture (85) | Beef | Sequencing of V6 to V8 regions of 16S rRNA segments^i^ | 74.95 | | | | 11.72 | |  |  |  |  |  |  |  |  | [16] |

^a^Primer (name): forward (Arc915af), 5′-AGGAATTGGCGGGGGAGCAC-3′; reverse (Arc1386R), 5′-GCGGTGTGTGCAAGGAGC-3′ [13]

^b^Primer [conserved amino acid sequence element targeted]: forward [FGGSQR], 5′-TTCGGTGGATCDCARAGRGC-3';

reverse [GFYGYDL], 5′-GBARGTCGWAWCCGTAGAATCC-3' [18]

^c,d^Primer (name): forward (Met86F), 5’-GCTCAGTAACACGTGG-3’; reverse (Met471R), 5′-GWRTTACCGCGGCKGCTG-3′ [53, 54]

^e^Primer (name) [conserved amino acid sequence element targeted]: forward (mcrA-F) [FGGSQR], 5′-TTCGGTGGATCDCARAGRGC-3’;

reverse (mcrA-F) [GFYGYDL], 5′-GBARGTCGWAWCCGTAGAATCC-3’ [19]

^f^Primer (name): forward (Met86F), 5’-GCTCAGTAACACGTGG-3’; reverse (Met1340R), 5′-CGGTGTGTGCAAGGAG-3′ [54]

^g^Primer: forward, 5′- GGTGGTGTMGGATTCACACARTAYGCWACAG -3'; reverse, 5′- TTCATTGCRTAGTTWGGRTAGTT -3' [20]

^h^Primer (name): forward (Ar915aF), 5′-CCATCTCATCCCTGCGTGTCTCCGACTCAGTCAGGAATTGGCGGGGGAGCAC-3′;

reverse (Ar1386R), 5′-CCTATCCCCTGTGTGCCTTGGCAGTCTCAGNNNNNNNNNNNNCAGCGGTGTGTGCAAGGAGC-3′ [21]

^i^Generated via RNA-Seq

*Methanobrevibacter* species: *M. smi*, *Mbb. smithii*; *M. got*, *Mbb. gottschalkii*; *M. mil*, *Mbb. millerae*; *M. tha*, *Mbb. thaueri*; *M. rum*, *Mbb. ruminantium*; *M. oll*, *Mbb. olleyae*; *M. wol*, *Mbb. wolinii*; *M. woe*, *Mbb. woesei*; *M. sp*, *Methanobrevibacter sp.*; *Methanosphaera* species: *M. std*, *Msp. stadtmanae*; *Ms. sp*, *Methanosphaera* *sp.*; Other methanogen species: *Mcm*, *Methanocorpusculum*; *Thr*, *Thermoplasma*; Un, Unclassified methanogen.

**REFERENCES**

1. Seedorf, H., et al., *RIM-DB: a taxonomic framework for community structure analysis of methanogenic archaea from the rumen and other intestinal environments.* PeerJ, 2014. **2**.

2. Dereeper, A., et al., *Phylogeny.fr: robust phylogenetic analysis for the non-specialist.* Nucleic Acids Research, 2008. **36**(Web Server): p. W465-W469.

3. Lemoine, F., et al., *NGPhylogeny.fr: new generation phylogenetic services for non-specialists.* Nucleic Acids Research, 2019. **47**(W1): p. W260-W265.

4. Brosius, J., et al., *Complete nucleotide sequence of a 16S ribosomal RNA gene from Escherichia coli.* Proceedings of the National Academy of Sciences, 1978. **75**(10): p. 4801-4805.

5. Chakravorty, S., et al., *A detailed analysis of 16S ribosomal RNA gene segments for the diagnosis of pathogenic bacteria.* Journal of Microbiological Methods, 2007. **69**(2): p. 330-339.

6. Jin, W., et al., *Discovery of a novel rumen methanogen in the anaerobic fungal culture and its distribution in the rumen as revealed by real-time PCR.* BMC Microbiology, 2014. **14**(1).

7. Danielsson, R., et al., *Methane Production in Dairy Cows Correlates with Rumen Methanogenic and Bacterial Community Structure.* Frontiers in Microbiology, 2017. **8**.

8. Shi, W., et al., *Methane yield phenotypes linked to differential gene expression in the sheep rumen microbiome.* Genome Res, 2014. **24**(9): p. 1517-25.

9. Kittelmann, S., et al., *Two Different Bacterial Community Types Are Linked with the Low-Methane Emission Trait in Sheep.* PLOS ONE, 2014. **9**(7): p. e103171.

10. Auffret, M.D., et al., *Identification, Comparison, and Validation of Robust Rumen Microbial Biomarkers for Methane Emissions Using Diverse Bos Taurus Breeds and Basal Diets.* Frontiers in Microbiology, 2018. **8**.

11. Wallace, R.J., et al., *The rumen microbial metagenome associated with high methane production in cattle.* BMC Genomics, 2015. **16**: p. 839.

12. Stepanchenko, N., et al., *Microbial composition, rumen fermentation parameters, enteric methane emissions, and lactational performance of phenotypically high and low methane-emitting dairy cows.* Journal of Dairy Science, 2023. **106**(9): p. 6146-6170.

13. Zhou, M., E. Hernandez-Sanabria, and L.L. Guan, *Assessment of the Microbial Ecology of Ruminal Methanogens in Cattle with Different Feed Efficiencies.* Applied and Environmental Microbiology, 2009. **75**(20): p. 6524-6533.

14. Lopes, D.R.G., et al., *Assessing the relationship between the rumen microbiota and feed efficiency in Nellore steers.* Journal of Animal Science and Biotechnology, 2021. **12**(1).

15. Li, F. and L.L. Guan, *Metatranscriptomic Profiling Reveals Linkages between the Active Rumen Microbiome and Feed Efficiency in Beef Cattle.* Applied and Environmental Microbiology, 2017. **83**(9).

16. Tan, R.S.G., et al., *Identifying active rumen epithelial associated bacteria and archaea in beef cattle divergent in feed efficiency using total RNA-seq.* Current Research in Microbial Sciences, 2021. **2**.

17. Manafiazar, G., et al., *Association between fecal methanogen species with methane production and grazed forage intake of beef heifers classified for residual feed intake under drylot conditions.* Animal, 2021. **15**(8).

18. Denman, S.E. and C.S. McSweeney, *Development of a real-time PCR assay for monitoring anaerobic fungal and cellulolytic bacterial populations within the rumen.* FEMS Microbiology Ecology, 2006. **58**(3): p. 572-582.

19. Denman, S.E., N.W. Tomkins, and C.S. McSweeney, *Quantitation and diversity analysis of ruminal methanogenic populations in response to the antimethanogenic compound bromochloromethane.* FEMS Microbiology Ecology, 2007. **62**(3): p. 313-322.

20. Luton, P.E., et al., *The mcrA gene as an alternative to 16S rRNA in the phylogenetic analysis of methanogen populations in landfill.* Microbiology, 2002. **148**(11): p. 3521-3530.

21. Kittelmann, S., et al., *Simultaneous Amplicon Sequencing to Explore Co-Occurrence Patterns of Bacterial, Archaeal and Eukaryotic Microorganisms in Rumen Microbial Communities.* PLoS ONE, 2013. **8**(2).

22. Henderson, G., et al., *Rumen microbial community composition varies with diet and host, but a core microbiome is found across a wide geographical range.* Sci Rep, 2015. **5**: p. 14567.

23. Stewart, R.D., et al., *Assembly of 913 microbial genomes from metagenomic sequencing of the cow rumen.* Nature Communications, 2018. **9**(1).

24. Leahy, S.C., et al., *The Complete Genome Sequence of Methanobrevibacter sp. AbM4.* Standards in Genomic Sciences, 2013. **8**(2): p. 215-227.

25. Seshadri, R., et al., *Cultivation and sequencing of rumen microbiome members from the Hungate1000 Collection.* Nat Biotechnol, 2018. **36**(4): p. 359-367.

26. De Mulder, T., et al., *Exploring the methanogen and bacterial communities of rumen environments: solid adherent, fluid and epimural.* FEMS Microbiology Ecology, 2016.

27. Leahy, S.C., et al., *The Genome Sequence of the Rumen Methanogen Methanobrevibacter ruminantium Reveals New Possibilities for Controlling Ruminant Methane Emissions.* PLoS ONE, 2010. **5**(1).

28. Smith, P.H. and R.E. Hungate, *Isolation and characterization of Methanobacterium ruminantium n. sp.* J Bacteriol, 1958. **75**(6): p. 713-8.

29. Lee, J.-H., et al., *Methanobrevibacter boviskoreani sp. nov., isolated from the rumen of Korean native cattle.* International Journal of Systematic and Evolutionary Microbiology, 2013. **63**(Pt_11): p. 4196-4201.

30. Kelly, W.J., et al., *The complete genome sequence of the rumen methanogen Methanobrevibacter millerae SM9.* Standards in Genomic Sciences, 2016. **11**(1): p. 49.

31. Rea, S., et al., *Methanobrevibacter millerae sp. nov. and Methanobrevibacter olleyae sp. nov., methanogens from the ovine and bovine rumen that can utilize formate for growth.* International Journal of Systematic and Evolutionary Microbiology, 2007. **57**(3): p. 450-456.

32. Kelly, W.J., et al., *Draft Genome Sequence of the Rumen Methanogen Methanobrevibacter olleyae YLM1.* Genome Announcements, 2016. **4**(2).

33. Stewart, R.D., et al., *Compendium of 4,941 rumen metagenome-assembled genomes for rumen microbiome biology and enzyme discovery.* Nature Biotechnology, 2019. **37**(8): p. 953-961.

34. Gilbert, R., D. Ouwerkerk, and A. Klieve, *Archaeaphage Therapy to Control Rumen Methanogens*. 2011, Meat & Livestock Australia Limited: North Sydney.

35. Jeyamalar, J., *Investigation of rumen methanogens in New Zealand livestock : a thesis presented in partial fulfillment of the requirements for the degree of Doctor of Philosophy in Animal Science at Massey University, Palmerston North, New Zealand*, in *Animal Science*. 2010, Massey University: Palmerston North, New Zealand.

36. Gilmore, S.P., et al., *Genomic analysis of methanogenic archaea reveals a shift towards energy conservation.* BMC Genomics, 2017. **18**(1).

37. Kelly, W.J., et al., *The complete genome sequence of the rumen methanogen Methanobacterium formicicum BRM9.* Standards in Genomic Sciences, 2014. **9**(1).

38. Jarvis, G.N., et al., *Isolation and Identification of Ruminal Methanogens from Grazing Cattle.* Current Microbiology, 2000. **40**(5): p. 327-332.

39. Zhou, J., et al., *Correlation of Key Physiological Properties of Methanosarcina Isolates with Environment of Origin.* Applied and Environmental Microbiology, 2021. **87**(13).

40. Lambie, S.C., et al., *The complete genome sequence of the rumen methanogen Methanosarcina barkeri CM1.* Standards in Genomic Sciences, 2015. **10**(1).

41. Asakawa, S. and K. Nagaoka, *Methanoculleus bourgensis, Methanoculleus olentangyi and Methanoculleus oldenburgensis are subjective synonyms.* International Journal of Systematic and Evolutionary Microbiology, 2003. **53**(5): p. 1551-1552.

42. Battumur, U., et al., *Isolation and characterization of a new Methanoculleus bourgensis strain KOR-2 from the rumen of Holstein steers.* Asian-Australasian Journal of Animal Sciences, 2019. **32**(2): p. 241-248.

43. Paynter, M.J.B. and R.E. Hungate, *Characterization of Methanobacterium mobilis, sp. n., Isolated from the Bovine Rumen.* Journal of Bacteriology, 1968. **95**(5): p. 1943-1951.

44. Kelly, W.J., et al., *Complete Genome Sequence of Methanogenic Archaeon ISO4-G1, a Member of the Methanomassiliicoccales, Isolated from a Sheep Rumen.* Genome Announcements, 2016. **4**(2).

45. Li, Y., et al., *The complete genome sequence of the methanogenic archaeon ISO4-H5 provides insights into the methylotrophic lifestyle of a ruminal representative of the Methanomassiliicoccales.* Standards in Genomic Sciences, 2016. **11**(1).

46. Söllinger, A., et al., *Phylogenetic and genomic analysis of Methanomassiliicoccales in wetlands and animal intestinal tracts reveals clade-specific habitat preferences.* FEMS Microbiol Ecol, 2016. **92**(1).

47. Jin, D., et al., *Effects of dietary supplementation of active dried yeast on fecal methanogenic archaea diversity in dairy cows.* Anaerobe, 2017. **44**: p. 78-86.

48. Cersosimo, L.M., et al., *Influence of periparturient and postpartum diets on rumen methanogen communities in three breeds of primiparous dairy cows.* BMC Microbiology, 2016. **16**(1).

49. King, E.E., et al., *Differences in the Rumen Methanogen Populations of Lactating Jersey and Holstein Dairy Cows under the Same Diet Regimen.* Applied and Environmental Microbiology, 2011. **77**(16): p. 5682-5687.

50. Sirohi, S.K., et al., *The 16S rRNA and mcrA gene based comparative diversity of methanogens in cattle fed on high fibre based diet.* Gene, 2013. **523**(2): p. 161-166.

51. Seedorf, H., S. Kittelmann, and P.H. Janssen, *Few Highly Abundant Operational Taxonomic Units Dominate within Rumen Methanogenic Archaeal Species in New Zealand Sheep and Cattle.* Applied and Environmental Microbiology, 2015. **81**(3): p. 986-995.

52. Sollinger, A., et al., *Holistic Assessment of Rumen Microbiome Dynamics through Quantitative Metatranscriptomics Reveals Multifunctional Redundancy during Key Steps of Anaerobic Feed Degradation.* mSystems, 2018. **3**(4).

53. Cersosimo, L.M., et al., *Examination of the Rumen Bacteria and Methanogenic Archaea of Wild Impalas (Aepyceros melampus melampus) from Pongola, South Africa.* Microbial Ecology, 2014. **69**(3): p. 577-585.

54. Wright, A.-D.G. and C. Pimm, *Improved strategy for presumptive identification of methanogens using 16S riboprinting.* Journal of Microbiological Methods, 2003. **55**(2): p. 337-349.
